# Nociceptive Neurons Promote Myeloid-Derived Suppressor Cell Mobilization to Alleviate Post-Stroke Neuroinflammation

**DOI:** 10.7150/thno.119474

**Published:** 2025-08-11

**Authors:** Lingxin Cai, Jiayin Zhou, Xinran Cao, Huaping Huang, Qin Xie, Haifeng Chu, Gao Chen, Lulu Jin, Zhengwei Mao, Feng Yan

**Affiliations:** 1 Department of Neurological Surgery, The Second Affiliated Hospital of Zhejiang University School of Medicine, Hangzhou 310009, Zhejiang Province, China.; 2 Clinical Research Center for Neurological Diseases of Zhejiang Province, Hangzhou 310009, Zhejiang Province, China.; 3 Zhejiang Key Laboratory of Research and Transformation for Major Neurosurgical Diseases, Hangzhou 310009, Zhejiang Province, China.; 4 MOE Key Laboratory of Macromolecular Synthesis and Functionalization, Department of Polymer Science and Engineering, Zhejiang University, Hangzhou 310027, Zhejiang Province, China.; 5 State Key Laboratory of Transvascular Implantation Devices, Zhejiang Hangzhou 310009, China.

**Keywords:** nociceptive neurons, myeloid-derived suppressor cells, neuroinflammation, bone marrow, stroke

## Abstract

**Background:** Bone marrow serves as a source and reservoir of immune cells and plays a critical role in central nervous system diseases. Nociceptive neurons are widely distributed in the bone marrow, but their post-infarction changes and immunological functions remain largely unexplored.

**Methods:** A mouse model of middle cerebral artery occlusion (MCAO) and human skull bone marrow samples from stroke patients were analyzed. Calcitonin gene-related peptide (CGRP) signaling was manipulated via receptor knockout and targeted nanoparticle delivery. Immune responses were evaluated primarily through flow cytometry, immunofluorescence, and single-cell RNA sequencing.

**Results:** Activation of nociceptive neurons after cerebral infarction promoted myeloid-biased hematopoiesis in the bone marrow and increased infiltration of myeloid cells into brain tissue, resulting in anti-inflammatory and neuroprotective effects. This regulatory mechanism was mediated by CGRP, which enhanced the proliferation and mobilization of downstream myeloid-derived suppressor cells (MDSC), ultimately improving stroke outcomes. To overcome the hypotensive side effects of CGRP, we employed aged neutrophil membrane-coated nanoparticles for its targeted delivery to bone marrow, achieving sustained release and enhanced efficacy.

**Conclusion:** Nociceptive neurons critically modulate post-stroke bone marrow immune responses by releasing CGRP and activating MDSC. Targeted CGRP delivery to bone marrow represents a promising strategy to suppress neuroinflammation and improve neurological recovery after cerebral infarction.

## Introduction

Cerebral infarction is an acute cerebrovascular disease with high incidence, disability, and mortality rates, caused by the obstruction of cerebral blood vessels, leading to local brain tissue ischemia and hypoxia, subsequently resulting in cell death and dysfunction 1. Immune cells in acute-phase neuroinflammation play a role in debris clearance and tissue repair 2. However, excessive or indiscriminate immune activation may lead to secondary damage**,** adversely affecting the prognosis 3. Therefore, establishing an immune environment conducive to tissue repair is crucial for promoting neurological recovery after ischemic stroke.

In recent years, increasing attention has been given to the role of the brain's peripheral immune niche in which bone marrow as the source and reservoir of immune cells is considered to play a key role in shaping the brain's immune microenvironment and responding to disease 4-7. In response to stress conditions such as tissue injury, hemorrhage, and inflammation, acute hematopoietic changes occur in the bone marrow, accompanied by the secretion of various cytokines 8-9. For instance, in a mouse model of multiple sclerosis, the bone marrow mobilizes neutrophils and exacerbates demyelination 10. However, in the case of acute cerebral hemorrhage, the bone marrow mobilizes Ly6C^low^ monocytes to suppress neuroinflammation and improve the prognosis 11. The results of single-cell RNA sequencing revealed that systemic bone marrow cells in mice exhibit a myeloid differentiation bias during the acute phase of cerebral infarction 6. However, the specific changes in immune cell types, their recruitment to the lesion and their role in stroke remain unclear.

It is currently believed that the neural signals with rapid, widespread, and persistent effects are related to bone marrow immune response after cerebral infarction 12. Previous studies have indicated that the sympathetic nervous system plays a role in bone marrow mobilization 13. However, there are more extensively distributed nociceptive neurons in the bone marrow cavity 14, which are a specialized class of afferent nerve fibers capable of detecting harmful stimuli such as temperature, chemicals, and inflammatory mediators 15-16. Their secretion of the primary neurotransmitter calcitonin gene-related peptide (CGRP) can act on immune cells with receptor activity-modifying protein 1 (RAMP1), ultimately promoting hematopoiesis 17-18. In addition, increasing evidence suggests that nociceptive neurons play a significant role in immune modulation 19. After peripheral tissue injury, the CGRP released by activated nociceptive neurons regulates neutrophils and monocytes to promote wound healing 20. In the melanoma tumor microenvironment, neurons release CGRP to promote T cell exhaustion and inhibit anti-cancer immune responses 21-22 In infectious diseases, nociceptive neurons release neuropeptides that modulate the activity of adaptive immune cells, enhancing the host's defense capabilities 23. However, the activation of nociceptive neurons after cerebral infarction and their impact on bone marrow immune cells have not been studied.

This study aims to investigate the role of nociceptive neuron activation in bone marrow response following cerebral infarction and the effects of downstream immune cell changes on cerebral infarction prognosis, with validation in human bone marrow samples. Additionally, CGRP will be delivered to the bone marrow using biomimetic membrane nanoparticles to target the marrow, eliminating the hypotensive side effects, while enhancing efficacy and prolonging the drug's half-life.

## Methods

### Study design

To investigate the role of bone marrow nociceptive neurons in post-stroke neuroinflammation, we integrated murine models and human clinical samples. Human skull bone marrow was collected from 6 patients undergoing decompressive craniectomy for massive cerebral infarction (within 48 h post-onset) and 5 controls (hemifacial spasm patients). These samples were processed to validate myeloid-biased hematopoiesis after cerebral infarction. For functional validation, human bone marrow cells were cultured *in vitro* with CGRP and CGRP-NM@NPs to assess MDSC proliferation and anti-inflammatory effects. Confocal fluorescence images were used to assess nociceptive neuron activation. To validate CGRP-mediated regulation, RAMP1 knockout mice were generated via bone marrow transplantation. Single-cell RNA sequencing of bone marrow immune cells identified MDSC as key downstream effectors, with flow cytometry and Gr-1 antibody depletion confirming their functional role. To overcome hypotensive side effects of CGRP, we synthesized aged neutrophil membrane-coated nanoparticles (CGRP-NM@NPs) for targeted bone marrow delivery. Human bone marrow samples and biomimetic nanoparticle validation were integrated to support translational potential.

### Animal studies

Male C57BL/6 mice (8-10 weeks old, weighing 21-25g) were used for *in vivo* experiments. All animal procedures were approved by the Ethics Committee of the Second Affiliated Hospital of Zhejiang University and conducted in accordance with the National Institutes of Health (NIH) Guide for the Care and Use of Laboratory Animals (NIH Publication No. 80-23, revised 1996), ensuring full compliance with animal welfare and ethical standards. The animals were housed in a controlled environment with regulated temperature and humidity under a 12h light/dark cycle, with free access to food and water. Mice were randomly assigned to either the sham surgery group or the MCAO group. All treatments and analyses were performed in a blinded manner whenever feasible to ensure objectivity. All experiments involving animals were authorized by the Ethics Committee of the Second Affiliated Hospital of Zhejiang University (Approval No. AIRB-2021-607).

### MCAO model induction

Permanent MCAO was induced in mice using a silicone-coated nylon monofilament (RWD Life Science, China). Mice were anesthetized with isoflurane (induction: 4%, maintenance: 1.5-2%) in a mixture of 30% O_2_ and 70% N_2_, with continuous monitoring of physiological parameters including body temperature, heart rate, and respiratory rate. A midline neck incision exposed the left common carotid artery, and the external carotid artery was ligated. A monofilament was inserted into the internal carotid artery to block the middle cerebral artery, with real-time cerebral blood flow monitored using a laser Doppler flowmeter (moorVMS-LDF2, Moor Instruments, UK) to ensure successful occlusion (reduction to < 20% of baseline). The incision was sutured after filament placement. Sham-operated mice underwent identical procedures without filament insertion 24.

### Human sample collection

Bone marrow samples were collected from the skull of 6 patients undergoing decompressive craniectomy for massive cerebral infarction and 5 control patients undergoing facial nerve decompression for hemifacial spasm. The clinical characteristics and basic information of recipients were collected from the hospital case system (Table S1). All samples were obtained within 48 h post-onset. Inclusion criteria were age >18 years and absence of major systemic diseases (e.g., heart failure, liver disease, severe infection, cancer, autoimmune disorders, or hematologic diseases). Skull fragments were collected using bone rongeurs, and bone marrow cells were isolated by saline. All procedures were approved by the Ethics Committee of the Second Affiliated Hospital of Zhejiang University (Approval No. 2024-0978) with informed consent from patients or their legal representatives. Remaining bone fragments were stored in liquid nitrogen for further experiments.

### Human bone marrow cell processing and flow cytometry

Bone marrow cell suspensions were filtered through a 300-mesh sieve to remove bone fragments and tissue, followed by red blood cell lysis to obtain a single-cell suspension of approximately 5×10^6^ cells in 100 µL. Cells were incubated with 1 µg CD16/CD32 blocking antibody at room temperature for 20 min, followed by staining with Zombie viability dye. Surface staining was performed using fluorochrome-conjugated antibodies (CD11b, CD14, CD15, HLA) at room temperature for 20 min in the dark. For nuclear membrane permeabilization, cells were treated with 1 mL FOXP3 Fix Buffer, vortexed, and incubated at room temperature for 20 min in the dark. After centrifugation, cells were washed with 1 mL FOXP3 Perm buffer, resuspended, and incubated for 15 min in the dark. The pellet was resuspended in 100 µL FOXP3 Perm buffer, and 1 µL nuclear KI67 antibody was added for 30 min. After washing, cells were resuspended in flow cytometry buffer and analyzed using a CytoFLEX flow cytometer (Beckman Coulter, Brea, CA, USA). Data were analyzed with Cytexpert Software Version 2.4.0.28. Reagent details are provided in the Table S2.

### *In vitro* culture of human cells

Skull fragments were obtained using bone rongeurs and incubated in 5 mL PBS with shaking for 10 min. Bone fragments and soft tissues were removed by filtration, and cells were collected by centrifugation at 600 g for 5 min. Cell count and viability were assessed using trypan blue staining. Cells were resuspended in complete medium (RPMI 1640, 10% FBS, 1% penicillin-streptomycin) and seeded at 10^6^ cells per T25 flask in 5 mL medium. Cell suspension and morphology were confirmed under a microscope. Cells were treated with CGRP according to experimental groups, and medium was replaced with fresh CGRP-containing medium on the second day. On day 3, cells were harvested and processed for flow cytometry as described above.

### Ablation and stimulation of nociceptive neurons

Mice were injected with escalating doses of resiniferatoxin (RTX, Sigma-Aldrich, 30, 70, and 100 µg kg⁻¹) for 3 consecutive days, while control littermates received vehicle injections. Nociceptive neurons were ablated within 3-4 weeks. Mice were fed a capsaicin-containing diet (100 ppm, 100 mg kg⁻¹) or a control diet for 1 week to stimulate nociceptive neurons 17.

### Fluorescence staining and image analysis of mouse bone tissue

Mouse skulls were dissected, and the dura mater was removed. After washing and drying, bones were fixed in 4% PFA at 4 °C overnight. Bones were decalcified in 10 mL EDTA at 4 °C for 1 week, with EDTA replaced every 3 days until soft. Decalcified bones were washed with PBS, blocked with 20% donkey serum (Sigma) in 0.5% Triton X-100/PBS at 4 °C for 24 h, and incubated with anti-CGRP (D5R8F, Cell Signaling) at 4 °C overnight. After PBS washing, samples were incubated with donkey anti-rabbit IgG AF488 (A32790, Invitrogen) at 4 °C for 48-72 h, followed by DAPI staining for 5 min. Bones were cleared with RapiClear 1.52 at 4 °C overnight and imaged using a confocal microscope. For leg bones, samples were fixed in 4% PFA, decalcified in EDTA for 1 week, and cryoprotected in 30% sucrose/4% PFA at 4 °C overnight. Sections (10 µm) were blocked with 5% BSA for 1 h, incubated with anti-CGRP at 4 °C overnight, and stained with donkey anti-rabbit IgG AF488 for 1 h at room temperature. Nuclei were counterstained with DAPI, and images were acquired using a Leica DMI8 confocal microscope. Image analysis was performed using ImageJ.

### Transgenic mice and bone marrow transplantation

Wild-type (WT) mice were injected intravenously with busulfan (25 mg kg^-1^) for 4 consecutive days to ablate hematopoietic stem cells (HSCs). Bone marrow cells were isolated from RAMP1 knockout (RAMP1^⁻/⁻^) mice and transplanted into busulfan-treated WT mice via tail vein injection (5×10^6^ cells per mouse) 68. At 28 days post-transplantation, HSC engraftment was verified by flow cytometry, and chimerism was confirmed by RT-qPCR. BMT mice were then used for cerebral infarction model experiments.

### Single-cell RNA sequencing data analysis

Single-cell RNA sequencing data were processed and analyzed using Seurat R package (v5.0). Quality control was performed by removing low-quality cells (UMI counts < 400 or > 4000) and cells with high mitochondrial gene content (> 5%). Data normalization and scaling were conducted using the "NormalizeData" and "ScaleData" functions, followed by batch effect correction. Highly variable genes were identified using "FindVariableFeatures", and dimensionality reduction was achieved through principal component analysis (PCA). Cell clustering was performed using Uniform Manifold Approximation and Projection (UMAP) with optimized parameters via "FindClusters". Differentially expressed genes were identified using "FindAllMarkers" or "FindMarkers". Cell type annotation was based on established marker genes from literature and CellMarker 2.0 database. For myeloid cell developmental trajectory analysis, we employed monocle R package (v2.34.0) to perform pseudotime analysis on highly variable genes. Cell trajectories were visualized using "plot_cell_trajectory" integrated with Seurat clustering results. Proliferation scores were calculated using Seurat's "AddModuleScore" function based on z-score normalized expression of proliferation-related genes. MDSC signature scores were computed similarly using published MDSC marker genes. Gene Ontology (GO) biological process analysis was performed to identify significantly enriched pathways in myeloid cell subsets.

### MDSC depletion

MDSC were depleted through intraperitoneal injection of anti-Gr-1 antibody (BioLegend, Cat# 400643). Specifically, 150 µL of αGr-1 antibody was administered 8 h prior to cerebral ischemia induction. Control mice received equivalent volumes of isotype IgG (BioLegend, Cat# 108436). Depletion efficiency was validated using flow cytometry and brain tissue immunofluorescence analysis.

### Neutrophil isolation and membrane extraction

Bone marrow cells were harvested from the tibias and femurs of mice, and neutrophils were isolated using density gradient centrifugation. Histopaque 1119 and Histopaque 1077 solutions were layered in a 15 mL centrifuge tube, followed by centrifugation at 1600 g, 25 °C for 30 min. The white cell layer at the 1.077-1.099 Percoll interface was collected, washed with cold PBS, and centrifuged at 600 g, 4 °C for 3 min to remove residual Percoll. Purified neutrophils were resuspended in complete medium (1640, fetal bovine serum, antibiotics) and cultured for 6 h to obtain aged neutrophils 25. Cell pellets were then subjected to three freeze-thaw cycles (-80 °C and 37 °C) and homogenized 20 times on ice. The homogenate was resuspended in lysis buffer (75 mM NaCl, 6 mM NaHCO₃, 1.5 mM KCl, 0.17 mM Na₂HPO₄, 0.5 mM MgCl₂, 20 mM HEPES, 1 mM EDTA, with protease inhibitors) and centrifuged at 3200 g, 4 °C for 5 min. The supernatant was ultracentrifuged at 21,000 g, 4 °C for 30 min to pellet the cell membrane, which was resuspended in distilled water and stored at 4 °C. Membrane protein concentration was determined using a BCA protein assay kit (Beyotime Biotechnology, Cat. P0010) 26.

### Nanoparticle preparation

Biomimetic membrane liposomes were prepared using the thin-film hydration method. A mixture of 20 mg soybean lecithin (Shanghai Yuanye Biotechnology), 5 mg cholesterol (Aladdin), and 10 mg PEG or CGRP-modified PEG (Xi'an Ruixi Biotechnology) was dissolved in chloroform and evaporated to form a thin film 27. The film was hydrated with PBS containing cell membrane suspension, maintaining a 1:1 mass ratio of phospholipid to membrane protein. The mixture was sonicated for 6 min in an ice bath and extruded multiple times through a polycarbonate membrane (100 nm pore size) to obtain uniform biomimetic membrane liposomes (NM@NPs). Each mouse received 100 µL of liposome solution via tail vein injection, ensuring consistent membrane protein and CGRP concentrations 28. Mouse status was monitored post-injection, and samples were collected at predetermined time points for analysis.

### Statistical analysis

Data are presented as mean ± standard deviation (SD) and analyzed using GraphPad Prism software (version 8.4.3). For normally distributed continuous variables, comparisons between two groups were performed using Student's t-test (two-tailed, assuming equal variance). Comparisons among multiple groups were conducted using one-way or two-way analysis of variance (ANOVA), followed by post hoc tests for pairwise comparisons where appropriate. For non-normally distributed data or small sample sizes (n < 6 per group), non-parametric tests (e.g., Mann-Whitney U test for two groups or Kruskal-Wallis test with Dunn's post hoc analysis for multiple groups) were applied. Statistical significance was defined as p < 0.05. All experiments were independently repeated at least three times, and sample sizes were chosen based on preliminary data to ensure adequate statistical power.

## Results

### Activation of nociceptive neurons in bone marrow after cerebral infarction reduces neuroinflammation and improves prognosis

**Figure 1:**
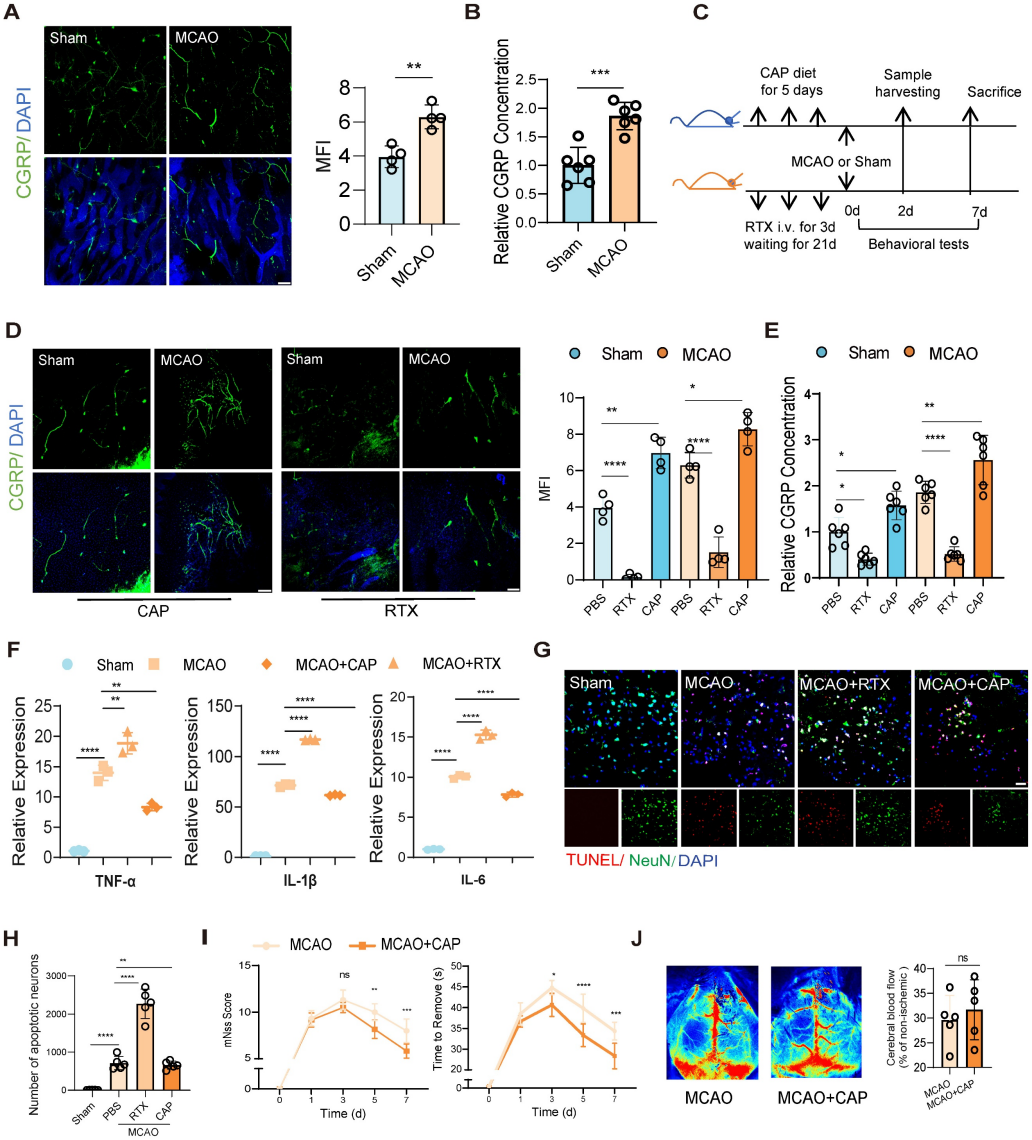
** Nociceptive neuron activation after cerebral ischemia attenuates neuroinflammation and improves outcomes.** (**A**) Confocal fluorescence images showing the activation of nociceptive neurons (green: CGRP^+^ neurons, blue: DAPI for nuclei) in the mouse skull 48 h after cerebral ischemia (n = 4). The scale bar is 100 µm. (**B**) CGRP levels in the bone marrow extracellular fluid measured by ELISA (n = 6). The results are presented as fold-change relative to the mean CGRP concentration in the bone marrow of untreated sham mice (set as 1.0 for each respective anatomical site: skull). (**C**) Experimental design for nociceptive neuron ablation using resiniferatoxin (RTX) and stimulation using capsaicin (CAP). (**D**) Representative confocal fluorescence images and quantification of nociceptive neurons to validate the success of interventions (n = 4). The scale bar is 100 µm. (**E**) CGRP levels in the bone marrow extracellular fluid measured by ELISA (n = 6). The results are presented as fold-change relative to the mean CGRP concentration in the bone marrow of untreated sham mice (set as 1.0 for each respective anatomical site: skull). (**F**) qPCR analysis of proinflammatory cytokine levels (TNF-α, IL-1β and IL-6) in ipsilateral brain tissues under conditions of nociceptive neuron stimulation or ablation (n = 3). (**G**) TUNEL staining to assess neuronal apoptosis in different groups (red: TUNEL, green: NenN, blue: DAPI for nuclei). The scale bar is 25µm. (**H**) Quantitative analysis of TUNEL staining results (n = 6). (**I**) Neurological deficits and functional outcomes evaluated by mNSS scores and adhesive removal tests at different time points after cerebral ischemia (n = 10). (**J**) Representative laser speckle contrast images showing cortical blood flow between MCAO and MCAO+CAP groups (n = 5). Quantification of relative CBF changes in the ischemic core and penumbra. All Data are shown as mean ± SD. Statistical significance was determined by ANOVA or Student's t test. *P < 0.05, **P < 0.01, ***P < 0.001, ****P < 0.0001.

To investigate post-stroke nociceptive neuron dynamics in bone marrow, we first employed confocal fluorescence imaging to observe significant activation of nociceptive neurons in the skull at 48 h post-infarction in a mouse model of middle cerebral artery occlusion (MCAO) (Figure 1A). And then we evaluated the concentration of CGRP in the bone marrow of mice, since CGRP is the primary neuropeptide released by nociceptive neurons, and it normally served as a well-established marker of neuronal activation. Our ELISA quantification revealed significantly elevated CGRP concentrations in bone marrow after MCAO (Figure 1B), revealing a parallel alteration with increased nociceptor activity. To further validate the functional role of nociceptive neurons, we employed resiniferatoxin (RTX) to ablate these neurons or capsaicin (CAP) to stimulate them 17 (Figure 1C). We then observed significantly higher activation of nociceptive neurons in the skull bone marrow of the CAP group compared to the group treated with PBS at 48 h post-infarction, regardless of whether the mice underwent MCAO surgery or not, while the RTX group exhibited significantly attenuated nociceptive neuron activation compared to PBS controls with or without MCAO surgery (Figure 1D).We further examined the CGRP concentration in different treated groups, and found significantly higher concentrations in the bone marrow of both skull and femur in the CAP group compared to the PBS group, regardless of MCAO surgery (Figure 1E). The RTX group exhibited an inverse pattern, with significantly lower concentrations compared to controls. The same experiments were replicated in the femur bone marrow, yielding consistent results (Figure S1A-D), indicating that nociceptive neuron activation is a pan-marrow phenomenon post-cerebral infarction, with consistent patterns observed across both cranial and appendicular skeletal compartments.

To delineate the implications of nociceptive neuron activation and its modulation on post-infarction outcomes, we conducted systematic assessments of neuroinflammation and cerebral infarction phenotypes. Reverse Transcription Quantitative PCR (RT-qPCR) analysis of tumor necrosis factor-alpha (TNF-α), interleukin-1 beta (IL-1β) and interleukin-6 (IL-6), three key neuroinflammatory cytokines, revealed that ablation of nociceptive neurons exacerbated neuroinflammation, whereas CAP stimulation significantly suppressed it (Figure 1F). TUNEL staining further demonstrated reduced neuronal apoptosis in the infarcted area of the CAP group, suggesting a neuroprotective effect of nociceptive neuron activation (Figure 1G-H). Behavioral assessments showed that the CAP group exhibited lower modified Neurological Severity Scores (mNSS) and shorter adhesive removal times, indicating significant functional recovery (Figure 1I). Additionally, speckle laser Doppler imaging revealed no significant changes in cerebral blood flow perfusion in either the RTX or CAP groups, suggesting that these interventions did not substantially alter blood flow dynamics (Figure 1J). These results are the first to focus on the immunomodulatory role of nociceptive neurons in the bone marrow following cerebral infarction and show that stimulating nociceptive neurons significantly alleviated neuroinflammation and improved the prognosis of neurological function.

### Nociceptive neurons release CGRP to influence myeloid-biased hematopoiesis

**Figure 2:**
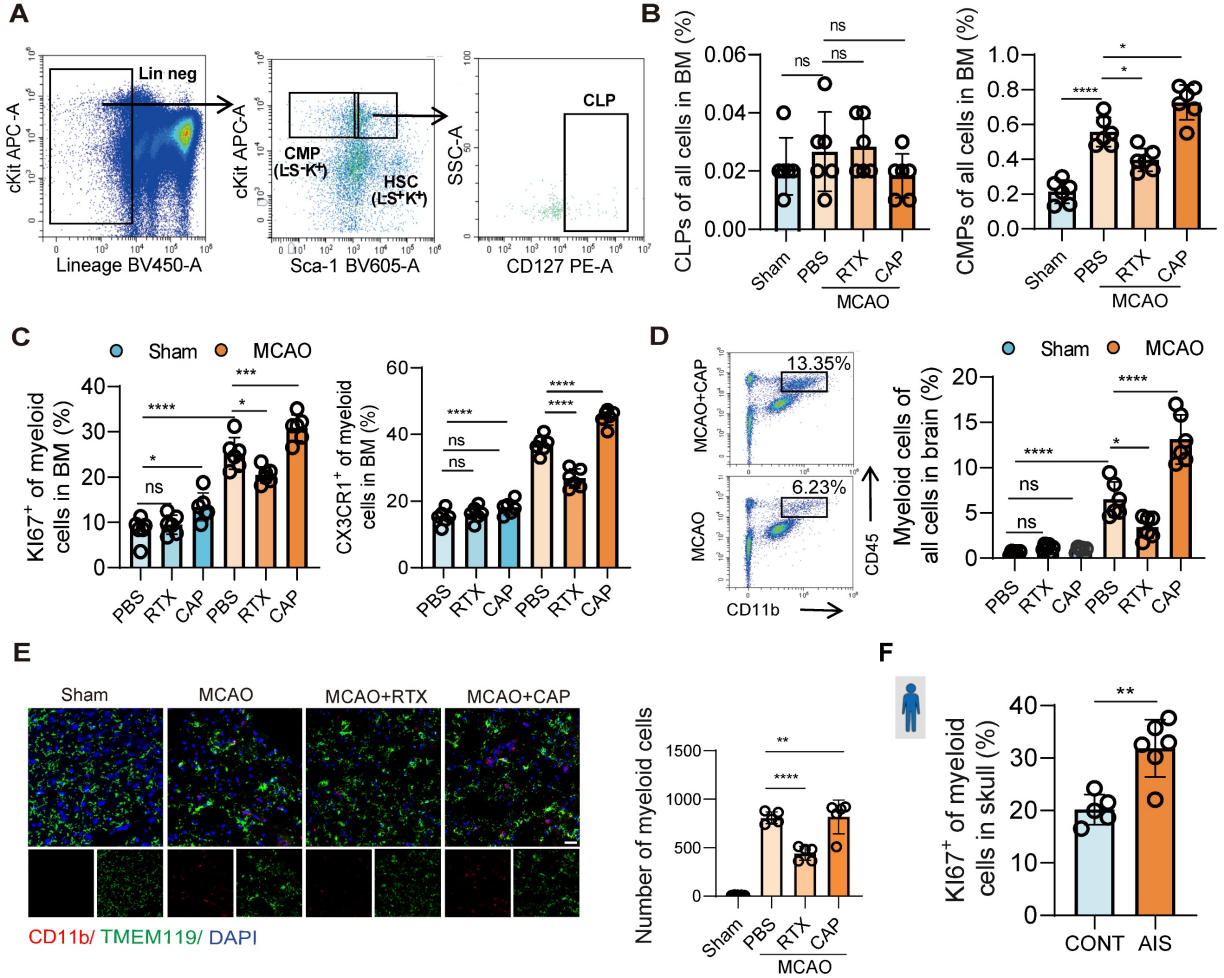
** Nociceptive neurons influence myeloid-biased hematopoiesis after cerebral ischemia.** (**A**) Gating strategy for common lymphoid progenitors (CLPs, Lin⁻Sca-1⁺c-Kit⁺IL-7Rα⁺) and common myeloid progenitors (CMPs, Lin⁻Sca-1⁻c-Kit⁺). (**B**) Flow cytometry analysis of CLPs and CMPs under conditions of nociceptive neuron ablation or stimulation, demonstrating myeloid-biased hematopoiesis (n = 6). (**C**) Flow cytometry analysis of KI67 and CX3CR1 expression in CD11b⁺ myeloid cells in the bone marrow, assessing proliferation and migration capabilities in different groups (n = 6). (**D**) Flow cytometry quantification of infiltrated CD11b⁺ myeloid cells in brain tissues (n = 6). (**E**) Immunofluorescence images showing infiltrated myeloid cells in brain tissues (red: CD11b, green: TMEM119, blue: DAPI for nuclei) (n = 6). The scale bar is 25 µm. (**F**) Flow cytometry analysis of human samples, showing increased KI67 positivity and enhanced proliferation of CD11b⁺ myeloid cells in the skull bone marrow of stroke patients (patients with AIS: n = 6; control subjects: n = 5). Data are shown as mean ± SD. Statistical significance was determined by ANOVA or Student's t test. *P < 0.05, **P < 0.01, ***P < 0.001, ****P < 0.0001.

Given the activation of nociceptive neurons post-infarction and their regulatory role in neuroinflammation, we further investigated their impact on bone marrow immune cells. A previous single-cell transcriptome sequencing study revealed that post-stroke, the bone marrow of the skull, femur, vertebrae, and other systemic sites exhibited consistent changes 6. Notably, our data further revealed parallel activation of nociceptive neurons in both skull and femur bone marrow post-infarction (Figure 1A, Figure S1A). Given the easy accessibility and systemic representativeness of femur bone marrow, this study focused on the femur for subsequent investigations to represent the changes in systemic bone marrow. Bone marrow cells are broadly categorized into myeloid and lymphoid lineages. To dissect post-infarction hematopoiesis dynamics, we quantified common myeloid progenitors (CMPs) and common lymphoid progenitors (CLPs) via flow cytometry. At 48 h post-infarction, CMPs were significantly elevated, whereas CLPs remained unchanged (Figure 2A-B). This myeloid-biased expansion aligns with prior sequencing data demonstrating increased bone marrow egress of LysM^+^ cells (predominantly myeloid lineage) in murine stroke models 6. Stimulation of nociceptive neurons further enhanced this myeloid-biased hematopoiesis (Figure 2A-B). To further confirm that the myeloid cells are sourced from bone marrow we performed flow cytometry to assess the proliferation and migration capacities of CD11b^+^ myeloid cells, and it turned out the expression of proliferation (KI67) and migration (CX3CR1) significantly increased, in which the CX3CR1 is the key chemokine that promote the migration capacity of immune cells to the infarcted region (Figure S2A). Results showed increased mobilization of myeloid cells in the bone marrow and enhanced infiltration of myeloid cells into the brain tissue post-infarction, with nociceptive neuron stimulation amplifying this trend (Figure 2C-D). Brain tissue fluorescence imaging further confirmed increased myeloid cell infiltration, particularly after nociceptive neuron stimulation (Figure 2E). To validate these findings in humans, bone marrow cells were isolated from skull samples of patients (6 from those undergoing decompressive craniectomy due to massive cerebral infarction and 5 controls from patients undergoing facial nerve decompression for hemifacial spasm). Flow cytometry analysis revealed significantly increased myeloid cell proliferation in the bone marrow of cerebral infarction patients (Figure 2F and Figure S2B). These results further underscored the critical role of nociceptive neurons in modulating the bone marrow's response to cerebral infarction, demonstrating their capacity to alleviate neuroinflammation through the promotion of myeloid-biased hematopoiesis within the bone marrow.

**Figure 3:**
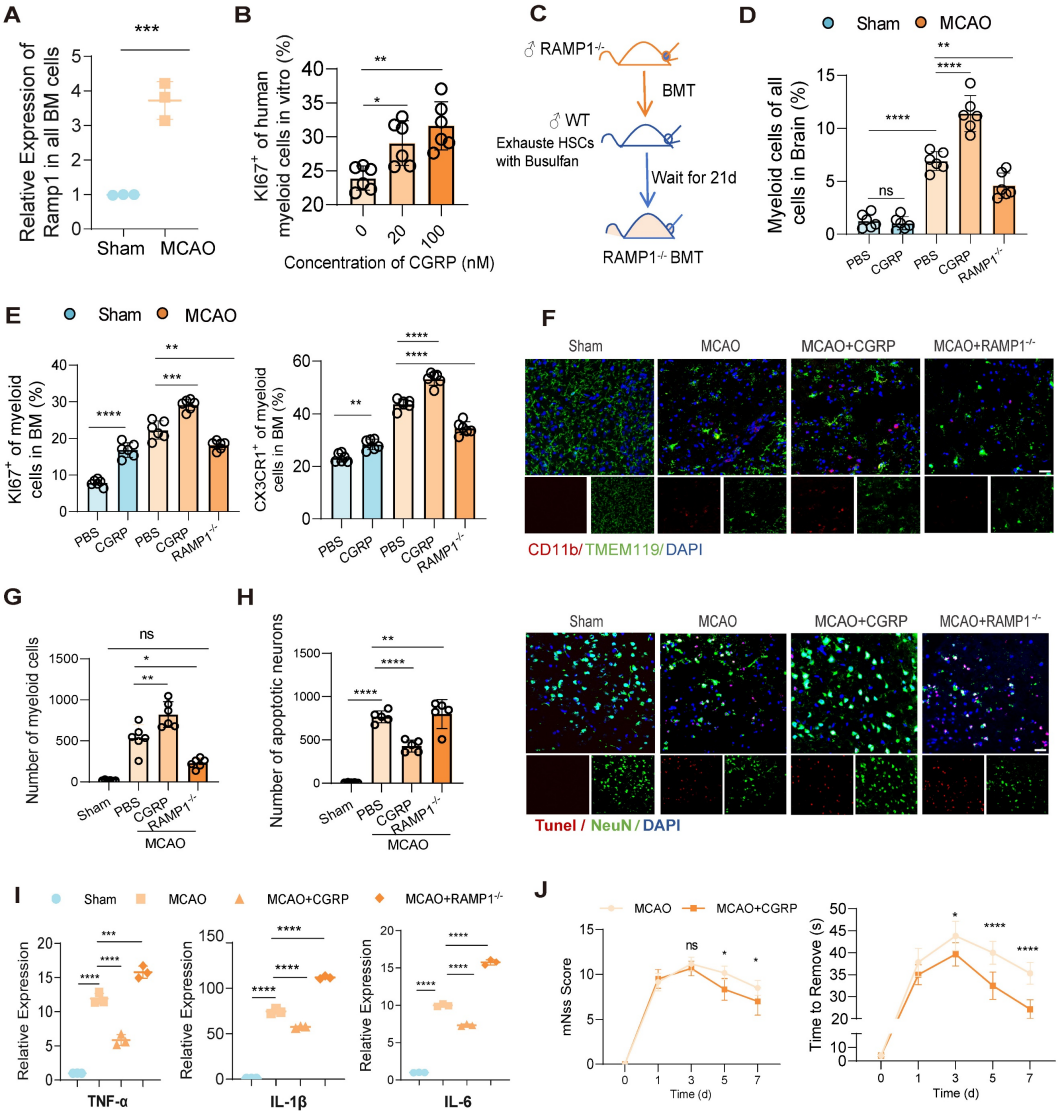
** Nociceptive neurons regulate bone marrow myeloid cell differentiation through CGRP.** (**A**) qPCR analysis of RAMP1 expression in bone marrow cells after cerebral ischemia (n = 3). (**B**) *In vitro* culture experiment of human skull bone marrow cells and flow cytometry analysis of KI67 expression in myeloid cells after CGRP treatment (n = 6). (**C**) Experimental design for bone marrow transplantation: ablation of bone marrow HSCs in wild-type (WT) mice using busulfan, followed by transplantation of bone marrow from RAMP1 knockout (RAMP1^-/-^) mice into WT mice to generate chimeric models. (**D**) Flow cytometry analysis of myeloid cells in the brain in sham and MCAO group (n = 6). (**E**) Flow cytometry analysis of KI67 and CX3CR1 expression in bone marrow myeloid cells to assess proliferation and migration capabilities in RAMP1 knockout mice and CGRP-treated groups (n = 6). (**F**)Immunofluorescence images showing infiltrated myeloid cells in brain tissues (red: CD11b, green: TMEM119, blue: DAPI for nuclei) (n = 6). The scale bar is 25 µm. (**G**) Quantitative analysis of myeloid cell infiltration staining results (n = 6). (**H**) Quantitative analysis of TUNEL staining results (n = 6) and immunofluorescence images showing neuronal apoptosis (red: TUNEL, green: NenN, blue: DAPI for nuclei). (**I**) qPCR analysis of TNF-α, IL-1β and IL-6 expression in brain tissues (n = 3). (**J**) Neurological deficits and motor coordination evaluated by mNSS scores and adhesive removal tests after CGRP treatment (n = 10). Data are shown as mean ± SD. Statistical significance was determined by ANOVA or Student's t test. *P < 0.05, **P < 0.01, ***P < 0.001, ****P < 0.0001.

In previous studies, nociceptive neurons have been shown to become activated within injured tissues and regulate myeloid cell function via the release of the neuropeptide CGRP 20, and the neurotransmitter CGRP exerts its effects through a receptor complex composed of the calcitonin receptor-like receptor and RAMP1 on immune cells 29. Our experiments demonstrated a parallel phenomenon revealing a significant upregulation of RAMP1 receptor expression in bone marrow cells from MCAO mice (Figure 3A). Therefore, we assume that the released CGRP may modulate bone marrow cell responses via RAMP1 receptors, and may enhance proliferation of myeloid cells. To validate our hypothesis, we examined whether elevated CGRP concentrations promote myeloid cell proliferation and whether RAMP receptors mediate this process. First, we isolated the human skull bone marrow cells and cultured these cells *in vitro* and treated them with different concentrations of CGRP for 48 h, and observed a concentration-dependent increase in human myeloid cells proliferation in these primary bone marrow cells (Figure 3B). And then to further investigate the regulatory role of CGRP/RAMP on bone marrow cells, we generated bone marrow-specific RAMP1 knockout mice using bone marrow transplantation (BMT) (Figure 3C). Wild-type (WT) mice were first treated with busulfan to ablate hematopoietic stem cells (HSCs), followed by transplantation of bone marrow from RAMP1^-/-^ mice. Flow cytometry confirmed successful ablation of HSCs after busulfan treatment, and bone marrow recovery was observed 28 days post-transplantation (Figure S3A). qPCR analysis confirmed that the majority of bone marrow cells were RAMP1^-/-^, with an estimated chimerism rate of approximately 80%, consistent with previous literature (Figure S3B). Post-infarction, RAMP1^-/-^ BMT mice exhibited decreased infiltration of myeloid cells into the brain tissue (Figure 3D) and reduced proliferation and mobilization of myeloid cells in the bone marrow (Figure 3E). These findings were corroborated by brain tissue immunofluorescence analysis (Figures 3F-G). Exogenous administration of CGRP enhanced these responses, with 20 µg identified as the optimal concentration (Figure S3C). Furthermore, CGRP administration in mice post-infarction alleviated neuronal apoptosis (Figure 3H), neuroinflammation (Figure 3I) and improved neurological functional outcomes (Figure 3J). Recent studies have increasingly highlighted the impact of CGRP on immune cells, and these results also demonstrated its influence on bone marrow myeloid cells after cerebral infarction and its role in mitigating neuroinflammation.

### CGRP regulates the mobilization of MDSC in the bone marrow

**Figure 4:**
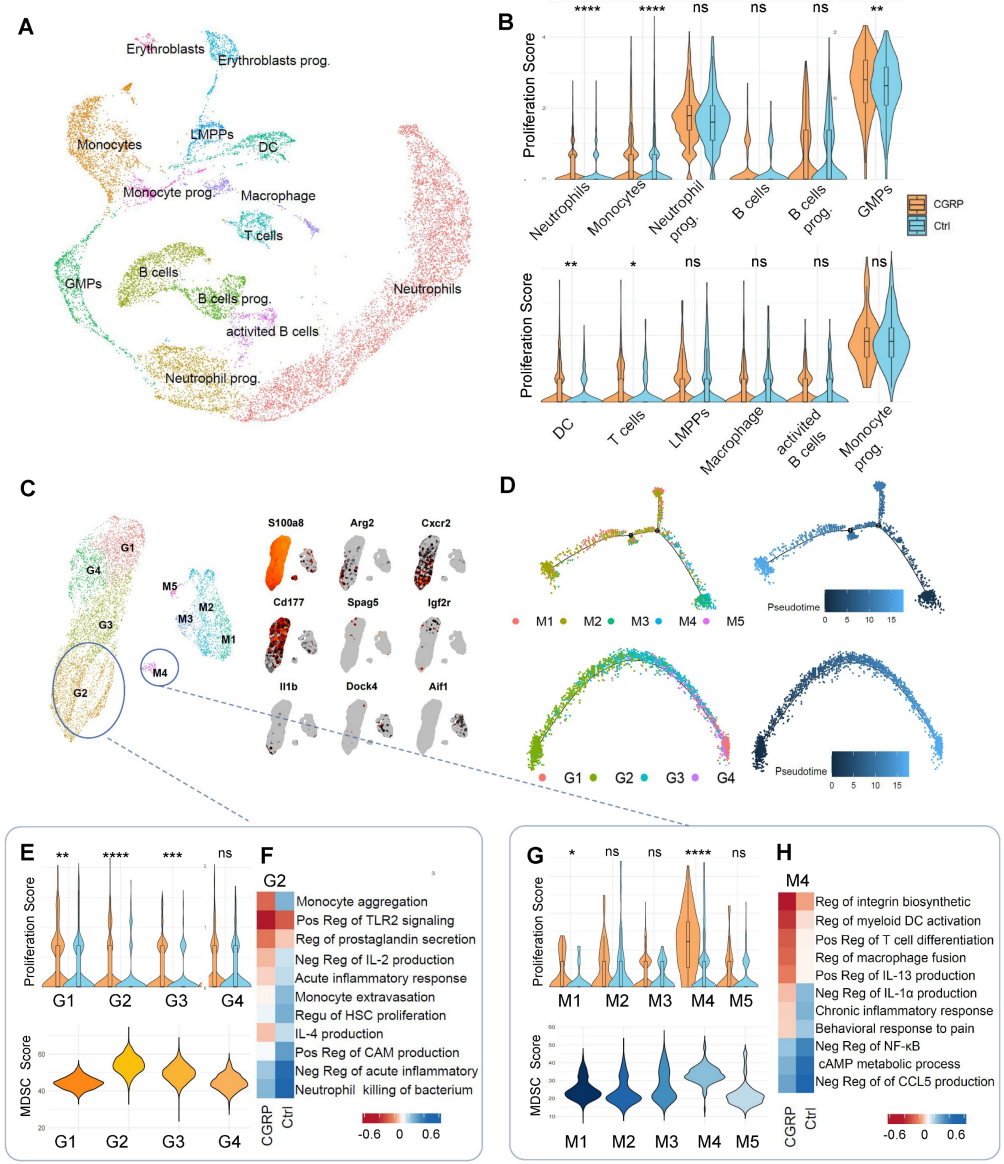
** Single-cell sequencing reveals CGRP-mediated regulation of MDSC proliferation and function.** (**A**) scRNA-seq analysis showing clustering of immune cells in the bone marrow after CGRP treatment, identifying 11 distinct cell populations. LMPP, Lymphoid-primed Multipotent Progenitors. GMPs, Granulocyte-Macrophage Progenitor. (**B**) Proliferation score analysis showing significantly enhanced proliferation of myeloid cells after CGRP treatment. (**C**) Re-clustering of myeloid cell subsets, defining 9 subpopulations, among which G2 and M4 exhibit characteristics of MDSC. (**D**) Pseudotime trajectory analysis showing that G2 and M4 are distributed at the starting point of the trajectory, suggesting their role as precursor cells of MDSC. (**E and F**) Proliferation score analysis of neutrophil subsets showing significantly enhanced proliferation of G2 after CGRP treatment, with the highest MDSC score and functional enrichment indicating enhanced anti-inflammatory and migratory capabilities. (**G and H**) Proliferation score analysis of monocyte subsets showing significantly enhanced proliferation of M4 after CGRP treatment, with the highest MDSC score and functional enrichment indicating enhanced anti-inflammatory, migratory, and hematopoietic proliferation capabilities. Data are shown as mean ± SD. Statistical significance was determined by Student's t test. *P < 0.05, **P < 0.01, ***P < 0.001, ****P < 0.0001.

To further investigate the impact of CGRP released by nociceptive neurons after cerebral infarction on bone marrow immune cells, we employed single-cell RNA sequencing (scRNA-seq) using the 10x Genomics Chromium platform. This approach allowed us to explore the molecular differences in bone marrow immune cells following CGRP treatment. We analyzed samples from control mice (9,155 cells) and CGRP-treated mice (9,060 cells) (n = 3), sequencing a total of 18,215 cells with an average sequencing depth of approximately 50,000 reads per cell. After quality control filtering to remove cells with low gene detection counts or high mitochondrial gene content, we used canonical correlation analysis (CCA) to identify 11 distinct cell clusters and classified cell types based on the expression of marker genes (Figure 4A and Figure S4A-C) 30-32. Proliferation scoring analysis revealed that CGRP treatment significantly enhanced the proliferation of myeloid cells (Figure 4B) 33. Further subclustering of myeloid cells defined nine subsets (four neutrophil-related and five monocyte-related clusters) (Figure 4C and Figure S4D). Among these, the G2 and M4 subsets exhibited hallmark genes highly similar to myeloid-derived suppressor cells (MDSC) (Figure 4C). Pseudotime trajectory analysis indicated that G2 and M4 subsets were located at the early stages of the trajectory (Figure 4D), suggesting they represent immature, less differentiated cell populations in the bone marrow rather than mature myeloid cells. This finding supports the possibility that G2 and M4 subsets may function as MDSC.

Following CGRP treatment, the proliferation scores of G2 and M4 subsets significantly increased. We therefore focused on these subsets and performed MDSC signature scoring, which confirmed their strong MDSC-like characteristics (Figure 4E-G) 34-36. Gene Ontology (GO) functional enrichment analysis further revealed that CGRP treatment enhanced their roles in suppressing inflammation, promoting monocyte migration, and supporting hematopoietic proliferation (Figure 4F-H). CGRP treatment significantly upregulated MDSC-related functional genes, further supporting the immunomodulatory and neuroprotective roles of these cells (Figure S4E). In summary, G2 and M4 represent distinct MDSC-like subpopulations. CGRP mainly promotes MDSC proliferation and mobilization and enhances their anti-inflammatory capacity.

**Figure 5:**
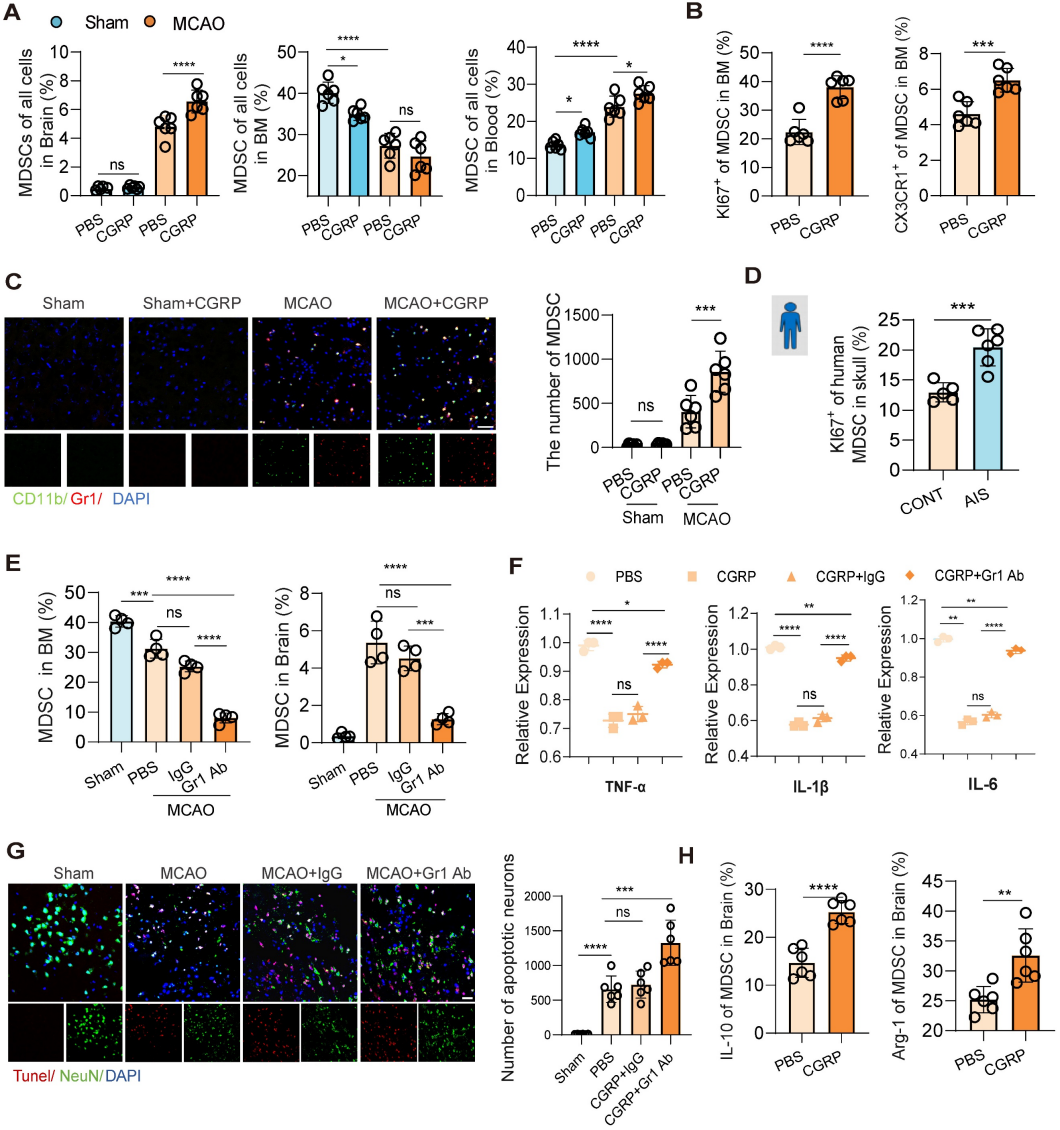
** CGRP exerts neuroprotective effects by regulating MDSC mobilization and function.** (**A**) Flow cytometry analysis of MDSC in brain tissues, bone marrow, and peripheral blood after cerebral ischemia and CGRP treatment (n = 6). (**B**) Flow cytometry analysis of KI67 and CX3CR1 expression in MDSC in the bone marrow (n = 6). (**C**) Immunofluorescence images showing increased infiltration of MDSC in the infarct area after CGRP treatment (green: CD11b^+^, red: Gr-1^+^ MDSC, blue: DAPI for nuclei) (n = 6), and quantitative analysis of MDSC cell infiltration staining results (n = 6). The scale bar is 25 µm. (**D**) Flow cytometry analysis of KI67 expression in MDSC in human skull bone marrow samples (patients with AIS: n = 6; control subjects: n = 5). (**E**) Flow cytometry validation of MDSC depletion using Gr-1 antibody in the bone marrow and brain tissues (n = 5). (**F**) qPCR analysis of TNF-α, IL-1β and IL-6 expression in brain tissues of cerebral ischemic mice after MDSC depletion and CGRP treatment (n = 3). (**G**) TUNEL staining to assess neuronal apoptosis in brain tissues (red: TUNEL, green: NenN, blue: DAPI for nuclei) (n = 6). The scale bar is 25 µm. (**H**) Flow cytometry analysis of of IL-10 and Arg-1 expression in MDSC in brain tissues of cerebral ischemic mice with or without CGRP treatment (n = 6). Data are shown as mean ± SD. Statistical significance was determined by ANOVA or Student's t test. *P < 0.05, **P < 0.01, ***P < 0.001, ****P < 0.0001.

To further validate that MDSC are downstream cells of CGRP released by nociceptive neurons, flow cytometry was used to assesse changes of MDSC populations in the bone marrow, peripheral blood, and brain tissue following cerebral infarction. The results showed that CGRP treatment reduced MDSC in the bone marrow while increasing their presence in the peripheral blood and brain tissue (Figure 5A and Figure S5A-B), while the enhanced expression of KI67 and CX3CR1 of MDSC in the bone marrow confirmed their proliferating and mobilizing capacities as well (Figure 5B). Immunofluorescence staining of brain tissue further confirmed a marked increase in MDSC infiltration in the infarcted region after CGRP treatment (Figure 5C). Similarly, in human skull bone marrow samples, we observed increased MDSC proliferation in patients with cerebral infarction (Figure 5D). Further analysis of MDSC subsets revealed that CGRP treatment significantly increased the population of monocytic MDSC (M-MDSC), while polymorphonuclear MDSC (PMN-MDSC) showed no significant changes (Figure S5C). To confirm their immunosuppressive function, we isolated MDSCs from the ischemic brain tissue of CGRP-treated mice using flow cytometric sorting and performed *in vitro* co-culture assays with T cells. The results demonstrated that these brain-infiltrating MDSCs effectively suppressed T cell proliferation (Figure S5D). These findings align with the single-cell RNA sequencing data, further supporting the role of CGRP in regulating MDSC proliferation and function. However, the specific roles and mechanisms of different MDSC subsets require further investigation.

To establish the critical role of MDSC in CGRP-mediated neuroprotection, we depleted MDSC using a Gr-1 antibody. Flow cytometry and immunofluorescence staining confirmed successful MDSC depletion, with a significant reduction in MDSC in the bone marrow and almost no infiltration in the brain tissue (Figure 5E and Figure S5E). Further flow cytometric analysis demonstrated that Gr-1 antibody treatment effectively reduced both major MDSC subsets, including M-MDSCs and PMN-MDSCs, in the bone marrow and brain, supporting the broad depleting efficacy of this strategy (Figure S5F). After MDSC depletion, the anti-inflammatory and neuroprotective effects of CGRP in cerebral infarction mice were markedly attenuated, as evidenced by increased expression of TNF-α, IL-1β and IL-6 (Figure 5F) and elevated neuronal apoptosis (Figure 5G). Higher levels of IL-10 and Arg-1 were also observed in MDSC in the brain of CGRP treated mice (Figure 5H). These results demonstrate that MDSC play a pivotal role in CGRP-mediated neuroprotection. In this section, we confirmed that CGRP induced MDSC proliferation and mobilization to the infarcted brain and exerted anti-inflammatory and neuroprotective effects. To minimize confounding effects from other CGRP-responsive immune cells, we performed GR-1 antibody-mediated MDSC depletion and it turned out that the neuroprotective effects of CGRP—including inflammation attenuation and apoptosis reduction—were significantly attenuated in the MCAO model following MDSC blockade. These findings demonstrate that post-stroke CGRP alleviates brain injury primarily by promoting MDSC infiltration in ischemic regions. Notably, residual neuroprotection persisted even after MDSC depletion, suggesting complementary roles of other CGRP-activated immune cell populations, which remains consistent with our proposed mechanism.

### CGRP-NM@NPs enable targeted bone marrow delivery

CGRP, as a potent vasodilator, induces a decrease in blood pressure (Figure S6A), which is particularly concerning during the acute phase of cerebral infarction, where maintaining stable blood pressure and cerebral perfusion is crucial for patient outcomes. Additionally, the short half-life of CGRP restricts its clinical efficacy 29. Given the critical role of CGRP in regulating neuroinflammation post-cerebral infarction and its potential for clinical translation, we developed a biomimetic membrane-based nanoparticle delivery system (CGRP-NM@NPs), which leverages the CXCR4-mediated bone marrow homing capability of senescent neutrophils to achieve targeted bone marrow delivery of CGRP 37, thereby avoiding its hypotensive side effects and enhancing its half-life and local concentration.

**Figure 6:**
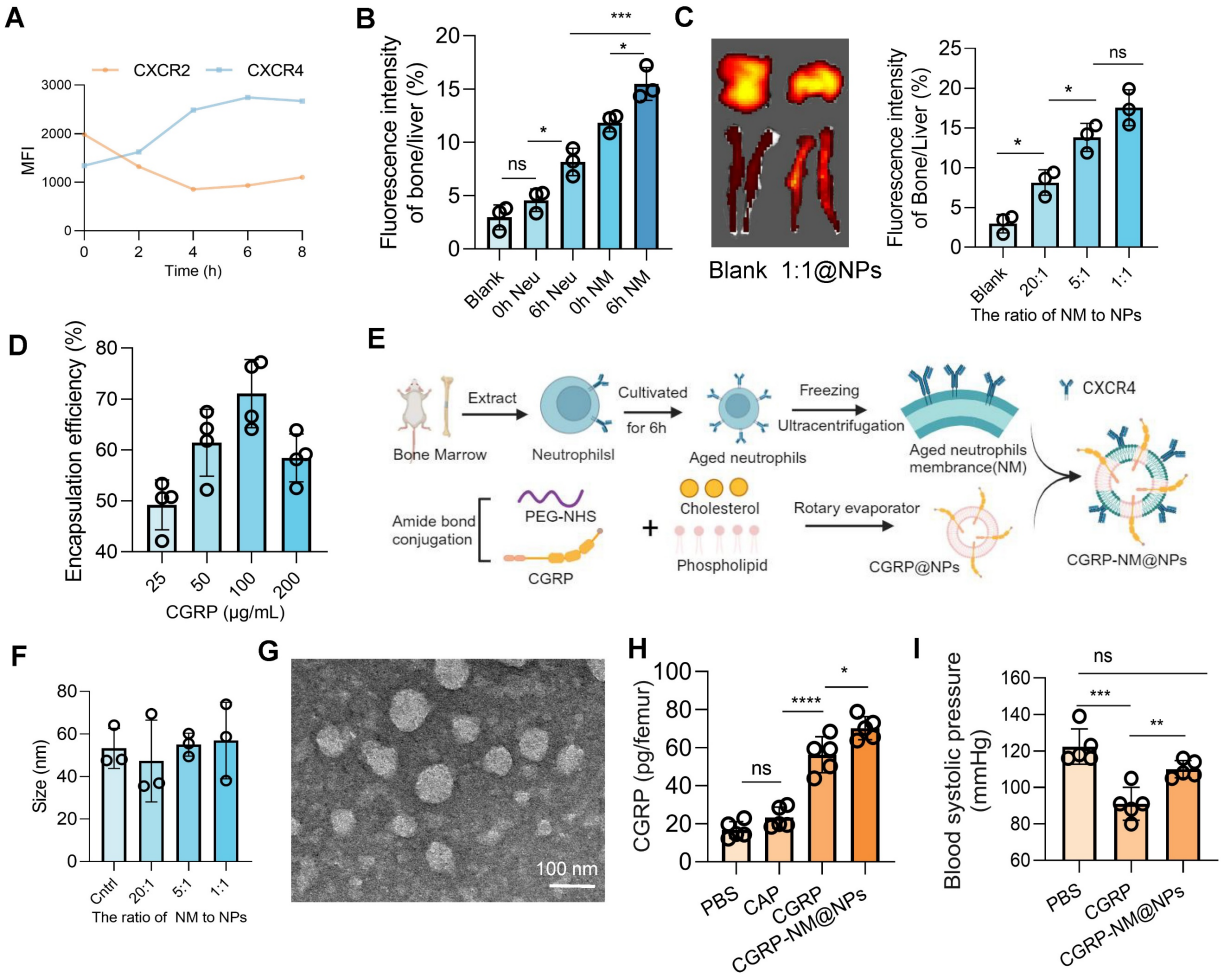
** Preparation and functional validation of nanoparticles for targeted bone marrow delivery of CGRP.** (**A**) Flow cytometry analysis showing peak expression of CXCR4 on neutrophils at 6 h (n = 5). (**B**) *In vivo* imaging to measure fluorescence intensity in the bone marrow and liver, showing optimal targeting efficiency of 6h neutrophil membrane (NM) (n = 3). (**C**) *In vivo* imaging to determine the optimal incorporation ratio of neutrophil membrane to nanoparticles (n = 3). (**D**) Ultraviolet fluorescence analysis to determine the optimal CGRP loading concentration, with the highest loading efficiency at 100 µg/mL (n = 4). (**E**) Schematic diagram of nanoparticle synthesis. (**F**) Size of nanoparticles with different membrane incorporation ratios (n = 3). (**G**) Transmission electron microscopy (TEM) images of the final CGRP-NM@NPs. (**H**) ELISA analysis showing the targeted delivery efficiency of CGRP-NM@NPs to the bone marrow (n = 4). (**I**) Blood pressure measurements showing that CGRP-NM@NPs do not cause a decrease in blood pressure after cerebral ischemic (n = 5). Data are shown as mean ± SD. Statistical significance was determined by ANOVA test. *P < 0.05, **P < 0.01, ***P < 0.001, ****P < 0.0001.

First, we analyzed the dynamic expression of CXCR4 using flow cytometry and found that CXCR4 expression peaked at 6 h, indicating optimal homing and targeting efficiency of neutrophils at this time point (Figure 6A). *In vivo* imaging further confirmed that neutrophils at the 6 h time point exhibited superior bone marrow targeting compared to those at 0 h, with enhanced targeting efficiency achieved using ultracentrifugation to isolate cell membranes, likely due to the concentrated effect of membrane components compared to intact cells (Figure 6B). By optimizing the ratio of neutrophil membranes to nanoparticles (Figure 6C) and the drug-loading capacity of CGRP (Figure 6D and Figure S6B), we successfully synthesized CGRP-NM@NPs (Figure 6E) with a CGRP loading capacity of 100 µg/ml and a 1:1 ratio of neutrophil membranes to nanoparticles. Dynamic light scattering (DLS) measurements demonstrated that CGRP-NM@NPs exhibited stable surface charge (Figure S6C-D) and uniform size distribution (Figure 6F and Figure S6E), with excellent stability under physiological conditions (Figure S6F), meeting the requirements for targeted delivery systems. Transmission electron microscopy (TEM) images confirmed the uniform morphology and consistent size distribution of the synthesized CGRP-NM@NPs (Figure 6G). ELISA assays revealed that CGRP-NM@NPs achieved significantly higher delivery efficiency to the bone marrow, increasing local drug concentration (Figure 6H). *In vivo* imaging further demonstrated the biodistribution pattern of CGRP-NM@NPs, with predominant accumulation in the liver, followed by the bone, and minimal distribution in other organs such as the spleen, lungs, and kidneys (Figure S6G). Moreover, in a mouse model of cerebral infarction, CGRP-NM@NPs did not induce a decrease in blood pressure, effectively avoiding the side effects associated with systemic CGRP administration (Figure 6I).

**Figure 7:**
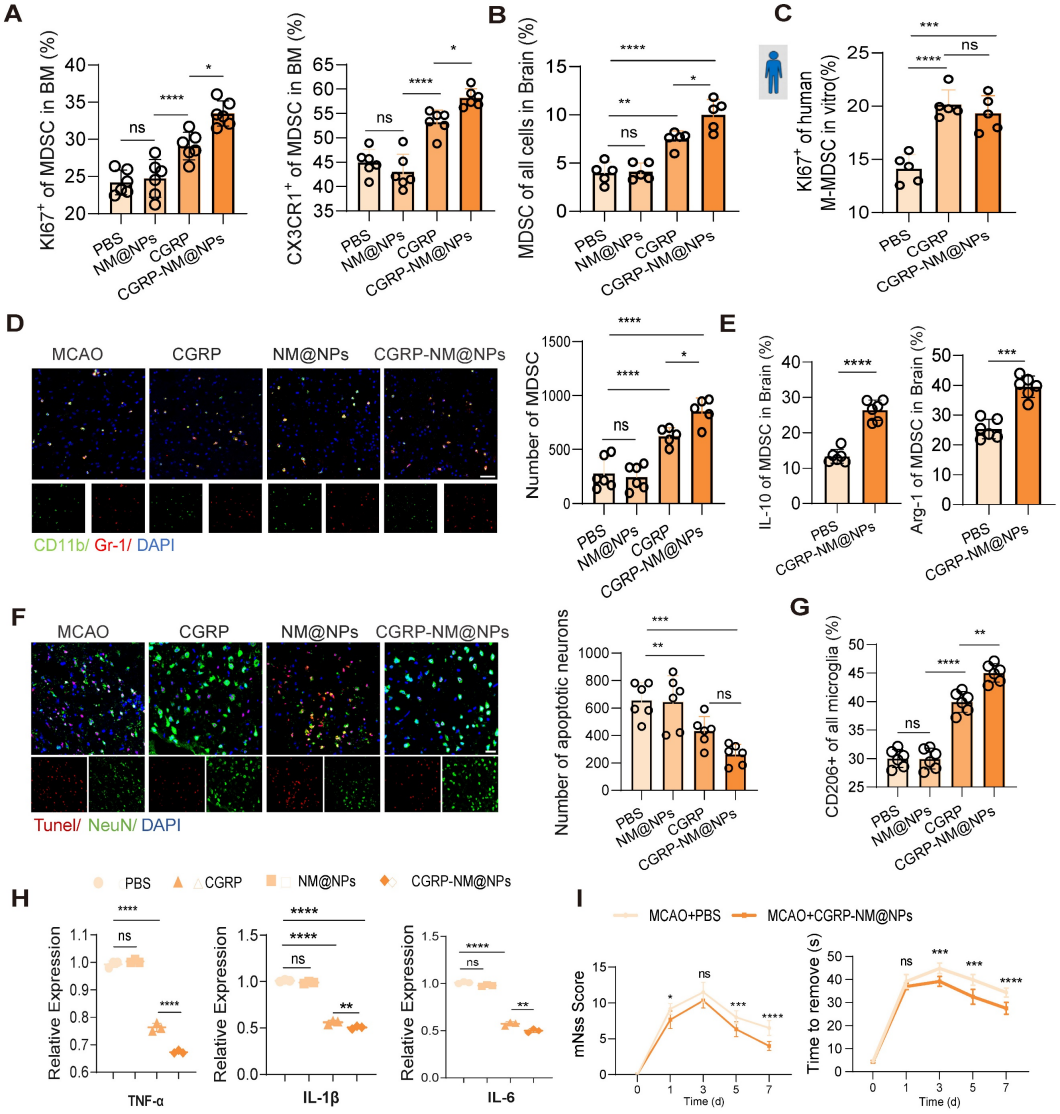
** Efficacy of CGRP-NM@NPs in suppressing neuroinflammation and promoting recovery after stroke.** (**A**) Flow cytometry analysis of KI67 and CX3CR1 to assess the proliferation and migration capacities of MDSC in the bone marrow with or without CGRP-NM@NPs treatment (n = 6). (**B**) Flow cytometry analysis of MDSC infiltration in brain tissues (n = 5). (**C**) *In vitro* culture experiment of human skull bone marrow cells to assess MDSC proliferation (n = 5). (**D**) Immunofluorescence images of MDSC infiltration in the infarct area of brain tissues (green: CD11b, red: Gr-1, blue: DAPI for nuclei) (n = 6). The scale bar is 25 µm. (**E**) Flow cytometry analysis of IL-10 and Arg-1 expression in MDSC (n = 6). (**F**) TUNEL staining to assess neuronal apoptosis in the infarct area (red: TUNEL, green: NenN, blue: DAPI for nuclei) (n = 6). The scale bar is 25µm. (**G**) Flow cytometry analysis of microglial polarization (n = 6). (**H**) qPCR analysis of TNF-α, IL-1β and IL-6 expression in brain tissues after CGRP-NM@NPs treatment (n = 3). (**I**) Neurological deficits and motor coordination evaluated by mNSS scores and adhesive removal tests (n = 10). Data are shown as mean ± SD. Statistical significance was determined by ANOVA or Student's t test. *P < 0.05, **P < 0.01, ***P < 0.001, ****P < 0.0001.

Finally, we validated the therapeutic efficacy of CGRP-NM@NPs in a mouse model of cerebral infarction. Treatment with CGRP-NM@NPs significantly enhanced the proliferative and migratory capacities of MDSC in the bone marrow, accompanied by increased MDSC infiltration in the brain tissue (Figure 7A-B). *In vitro* experiments using human skull bone marrow cells demonstrated that CGRP-NM@NPs treatment markedly promoted MDSC proliferation (Figure 7C), revealing its functional efficacy. Brain tissue fluorescence imaging further confirmed increased MDSC presence in the infarcted region (Figure 7D). Collectively, these results indicate that CGRP-NM@NPs effectively regulate the migration and function of MDSC from the bone marrow to the infarcted brain region, showing superior efficacy compared with CGRP alone. Additionally, CGRP-NM@NPs treatment enhanced the immunosuppressive function of MDSC, as evidenced by upregulated expression of IL-10 and Arg-1 (Figure 7E), elucidating the mechanism by which CGRP-NM@NPs exert neuroprotection through MDSC regulation. We further evaluated the therapeutic effects of CGRP-NM@NPs on cerebral infarction. TUNEL staining revealed a significant reduction in apoptotic neurons in the peri-infarct region of CGRP-NM@NPs-treated mice compared to controls (Figure 7F). Flow cytometry further demonstrated that CGRP-NM@NPs suppressed microglial polarization toward the M2 phenotype (Figure 7G) and qPCR analysis of brain tissue showed the anti-inflammatory efficacy of CGRP-NM@NPs (Figure 7H). Behavioral assessments showed that CGRP-NM@NPs treatment significantly improved neurological functional outcomes, as indicated by reduced mNSS and shorter adhesive removal times (Figure 7I).

To evaluate the safety of the synthesized nanoparticles, we conducted biochemical and pathological analyses on mice at 1, 3, and 7 days post-intravenous injection of CGRP-NM@NPs. Immunohistochemical results demonstrated that CGRP-NM@NPs treatment did not induce tissue damage or inflammatory responses in major organs, including the heart, liver, spleen, lungs, and kidneys (Figure S7A). Furthermore, no significant impact on liver or kidney function was observed (Figure S7B-C). In conclusion, this delivery system exhibits excellent biocompatibility and safety, as both the cell membrane and CGRP are endogenous biological components, further enhancing its potential for clinical translation.

## Discussion

Although significant progress has been made in the acute treatment of cerebral infarction through thrombolysis and thrombectomy, many patients still suffer from severe neurological deficits 38, highlighting the critical role of secondary neuroinflammation in the pathological progression of cerebral infarction 39. The bone marrow, as a crucial immune niche, has gradually garnered attention 40, yet its response mechanisms following cerebral infarction remain poorly understood. In this study, we discovered that nociceptive neurons are activated after cerebral infarction, and capsaicin stimulation of these neurons promotes CGRP release, driving myeloid-biased hematopoiesis in the bone marrow. Single-cell RNA sequencing revealed that MDSC play a central role in this process. Finally, we developed a bone marrow-targeted biomimetic membrane nanoparticle delivery system for CGRP, which effectively suppressed neuroinflammation and improved neurological functional recovery. These findings provide an in-depth exploration of bone marrow cell responses and neuro-immune interactions following cerebral infarction, demonstrating for the first time the pivotal role of nociceptive neurons in the brain-bone axis and achieving effective intervention. This study offers novel insights and directions for the development of therapeutic strategies for cerebral infarction.

In recent years, the interaction between the nervous and immune systems has garnered increasing attention 41-43. Nociceptive neurons, a class of widely distributed sensory neurons, not only play a critical role in perceiving noxious stimuli but also regulate the activity and function of immune cells through the release of neuropeptides such as CGRP 44-45. Numerous studies have explored the role of nociceptive neurons in immunity, revealing their immunomodulatory effects (primarily immunosuppressive) in contexts such as cancer, infectious diseases, and tissue injury 46-47. Compared to sympathetic nerves in peripheral tissues, nociceptive neurons are more densely distributed in the bone marrow 14, where they have been shown to significantly influence hematopoietic mobilization. Our study is the first to focus on the immunomodulatory role of nociceptive neurons in the bone marrow, and found these neurons significantly alter myeloid-biased hematopoiesis in the bone marrow after cerebral infarction. Notably, we observed significant activation of nociceptive neurons in both the skull and femur. Previous studies suggest that the bone marrow throughout the body responds synergistically under stress 48-49. A study published in *Cell* using sequencing data proposed that the bone marrow exhibits a consistent response pattern post-cerebral infarction, with the skull marrow potentially responding slightly earlier due to its proximity to the brain 6. However, other studies suggest that the skull marrow possesses unique immunological functions 50, and may play a special role in central nervous system diseases due to direct communication pathways between the skull and brain tissue 51-55. The differences between skull marrow and systemic bone marrow remain to be further elucidated. Nevertheless, when considering therapeutic targets and clinical translation, the systemic bone marrow is often regarded as a unified entity.

MDSC are a population of immature myeloid cells derived from bone marrow progenitors, which are typically mobilized from the bone marrow and migrate to damaged tissues under pathological conditions such as cancer, chronic inflammation, or infection 56-57. While sharing some characteristics with monocytes and neutrophils, MDSC exhibit distinct functional properties. They possess potent immunosuppressive capabilities, releasing molecules such as arginase-1 (Arg-1), inducible nitric oxide synthase (iNOS), and interleukin-10 (IL-10) to inhibit T cell activity and modulate macrophage polarization 58-59. However, their role in acute injury or inflammation is more complex. Studies have shown that MDSC can suppress excessive inflammatory responses and promote tissue repair in conditions such as acute liver injury and renal ischemia-reperfusion 60. Notably, exogenous transplantation of MDSC has been reported to alleviate neuroinflammation and improve neurological outcomes in the central nervous system diseases 61-63. In this study, single-cell RNA sequencing revealed that CGRP treatment enhanced MDSC-like subset proliferation in the bone marrow post-cerebral infarction. Flow cytometry and immunofluorescence confirmed CGRP-induced MDSC mobilization to the infarcted brain, where they exerted anti-inflammatory and neuroprotective effects. It is noteworthy that the anti-inflammatory effects of CGRP were significantly attenuated but not completely abolished after MDSC depletion, suggesting that MDSC play a critical but non-exclusive role in CGRP-mediated neuroinflammatory regulation. While CGRP modulates monocytes, macrophages, and T cells across various organ systems 64-65, its function in the bone marrow may differ due to its distinct immune microenvironment, enriched with immature cells and unique regulatory mechanisms 66-67. Moreover, different pathological signals, such as infection, cancer, or tissue injury, drive specific bone marrow responses 68-69. In cerebral infarction, the role of nociceptive neurons and their neurotransmitters may be disease-specific. Further studies using advanced single-cell RNA sequencing and other methodologies are needed to unravel the complex interactions between nociceptive neurons and bone marrow immunity.

Given the critical role of CGRP in regulating neuroinflammation following cerebral infarction, we developed a bone marrow-targeted nanoparticle delivery system, CGRP-NM@NPs, to overcome the limitations of systemic CGRP administration. As a potent vasodilator, systemic use of CGRP can lead to a decrease in blood pressure 70, which is particularly concerning during the acute phase of cerebral infarction, where maintaining stable blood pressure and cerebral perfusion is crucial for patient outcomes 71. Additionally, the short half-life of CGRP restricts its clinical efficacy 29. The targeted delivery of CGRP to the bone marrow via nanoparticles not only avoids its hypotensive side effects but also significantly enhances its half-life and local concentration. Our study demonstrated that CGRP-NM@NPs is more effective than CGRP alone in suppressing post-infarction neuroinflammation, and improving neurological functional recovery. In this study, the phenotypic observations were conducted at the 48h time point. Although neuroinflammation typically peaks around 72 h, interventions prior to the peak of inflammation are more effective in mitigating secondary brain damage and creating a favorable environment for neural repair 72. To strengthen the clinical relevance of our findings, we collected human skull samples, including six from patients undergoing decompressive craniectomy due to massive cerebral infarction and five controls from patients undergoing facial nerve decompression for hemifacial spasm. *In vitro* experiments using skull-derived bone marrow cells preliminarily validated the proposed mechanism: cerebral infarction induces myeloid-biased hematopoiesis in the bone marrow, and CGRP treatment enhances this bias while promoting the proliferation and mobilization of MDSC.

This study has several limitations. First, the activation of nociceptive neurons was primarily detected using confocal fluorescence imaging, which, while providing preliminary evidence, lacks comprehensive and dynamic observation capabilities. The role of nociceptive neurons in cerebral infarction and their specific functions in the bone marrow remain unexplored, and the precise mechanisms of their activation, temporal dynamics, and interactions with other immune cells require further investigation. Future studies could employ advanced techniques such as two-photon imaging or calcium imaging to monitor the activation dynamics of nociceptive neurons in real time, combined with genetic editing or pharmacological approaches to further dissect their regulatory mechanisms. Second, the mechanistic understanding of MDSC remains incomplete. Although single-cell RNA sequencing and functional experiments have preliminarily revealed the role of MDSC in cerebral infarction, the heterogeneity and functional complexity of MDSC warrant further exploration. For instance, MDSC are also present in the spleen 56, and the role of nociceptive neurons in splenic immune regulation has been reported. Therefore, additional tracing experiments or tissue-specific knockout models are needed to exclude potential confounding effects from splenic MDSC. Moreover, while we characterized MDSC using flow cytometry and functional markers such as IL-10, flow cytometry alone cannot fully distinguish MDSC from mature myeloid cells. Future studies could integrate more refined single-cell multi-omics technologies to further define the subpopulations of MDSC and elucidate their specific mechanisms in cerebral infarction. These limitations highlight directions for future research. By leveraging more advanced technologies and systematic experimental designs, we can further unravel the complex regulatory mechanisms of the nociceptive neuron-immune axis in the bone marrow and its therapeutic potential for central nervous system diseases.

Our results reveal the critical role of sensory neurons in the bone marrow's response to cerebral infarction, demonstrating that they promote the proliferation and mobilization of MDSC through the release of CGRP, thereby suppressing neuroinflammation and improving neurological functional recovery. Building on this mechanism, we developed a biomimetic membrane-based nanoparticle delivery system to achieve targeted bone marrow delivery of CGRP, offering a novel and translatable strategy for the treatment of cerebral infarction.

## Supplementary Material

Supplementary materials and methods, figures and tables.

## Abbreviations

CGRP: Calcitonin Gene-Related Peptide
MDSC: Myeloid-Derived Suppressor Cell
MCAO: Middle Cerebral Artery Occlusion
RTX: Resiniferatoxin
CAP: Capsaicin
CMP: Common Myeloid Progenitor
CLP: Common Lymphoid Progenitor
BMT: Bone Marrow Transplantation
RAMP1: Receptor Activity-Modifying Protein 1
ELISA: Enzyme-Linked Immunosorbent Assay
RT-qPCR: Reverse Transcription Quantitative Polymerase Chain Reaction
mNSS: Modified Neurological Severity Score
ANOVA: Analysis of Variance
GO: Gene Ontology
DLS: Dynamic Light Scattering
TEM: Transmission Electron Microscopy
NM@NPs: Neutrophil Membrane-Coated Nanoparticles
CGRP-NM@NPs: CGRP-Loaded Neutrophil Membrane-Coated Nanoparticles
PMN-MDSC: Polymorphonuclear MDSC
M-MDSC: Monocytic MDSC
PBS: Phosphate Buffered Saline
FBS: Fetal Bovine Serum
PFA: Paraformaldehyde
BCA: Bicinchoninic Acid
HSC: Hematopoietic Stem Cell
