# Supplementary material for: Nociceptive Neurons Promote Myeloid-Derived Suppressor Cell Mobilization to Alleviate Post-Stroke Neuroinflammation
Source: Theranostics. 2025 Aug 11;15(17):8897–915. doi: 10.7150/thno.119474 (PMC12439264; doi:10.7150/thno.119474)
Supplement: Supplementary file 1 — Supplementary materials and methods, figures and tables. [file thnov15p8897s1.pdf]

List of Supplementary Materials

Figure S1.Validation of nociceptive neuron activation in bone marrow

Figure S2.Gating strategy for flow cytometry

Figure S3.Validation analysis of CGRP-regulated bone marrow myeloid cells

Figure S4.Detailed analysis of single-cell sequencing and MDSC function validation

Figure S5.Validation of MDSC depletion and subset analysis

Figure S6.Characterization of nanoparticles

Figure S7.Safety validation of the nanoparticle

Tables S1.Human sample inclusion characteristic.

Tables S2.Antibodies used in flow cytometry.

Tables S3.Primers used for RT-qPCR.

Materials and Methods

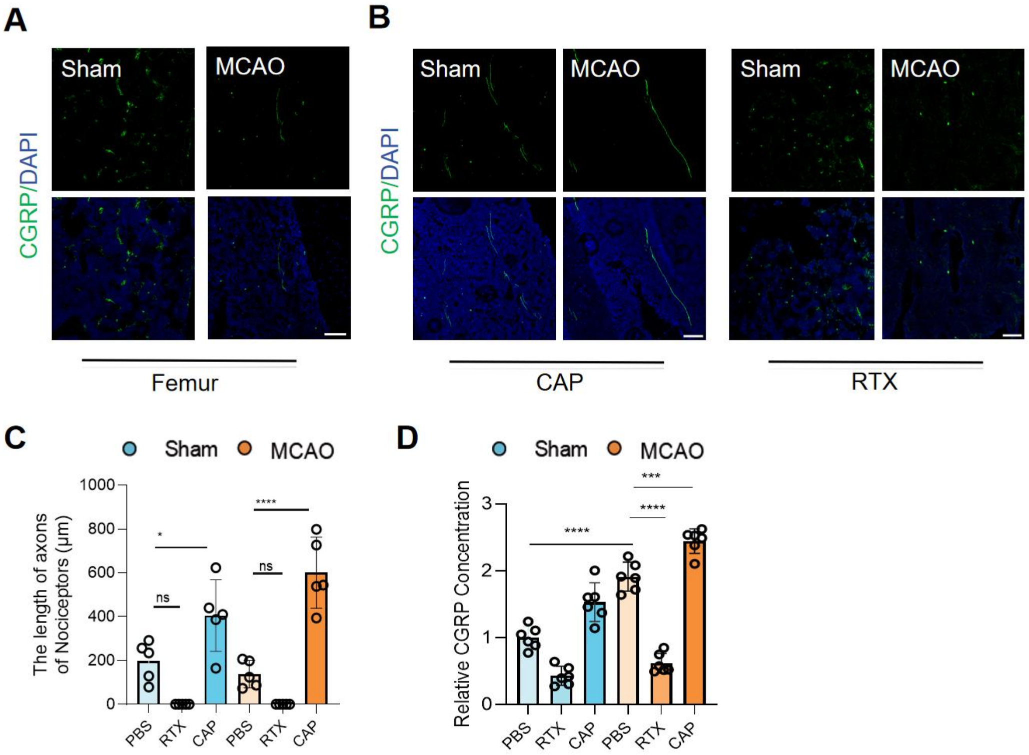

Figure S1. Validation of nociceptive neuron activation after cerebral ischemia and changes in bone marrow myeloid cells. (**A**) Confocal fluorescence images showing the activation of nociceptive neurons (green: $CGRP^+$ neurons, blue: DAPI for nuclei) in the femur of mice after cerebral ischemia. The scale bar is 50 μm. (**B**) Confocal fluorescence images showing changes in nociceptive neurons in the femur after CAP or RTX treatment. The scale bar is 50 μm. (**C**) Quantitative analysis showing significantly increased axonal length of nociceptive neurons in the femur after CAP treatment (n = 5). (**D**) CGRP levels in the bone marrow extracellular fluid (BMEF) measured by ELISA (n = 6). The results are presented as fold-change relative to the mean CGRP concentration in the bone marrow of untreated sham mice (set as 1.0 for each respective anatomical site: femur).Data are shown as mean ± SD. Statistical significance was determined by ANOVA or Student's t test. *P < 0.05, ****P < 0.0001.

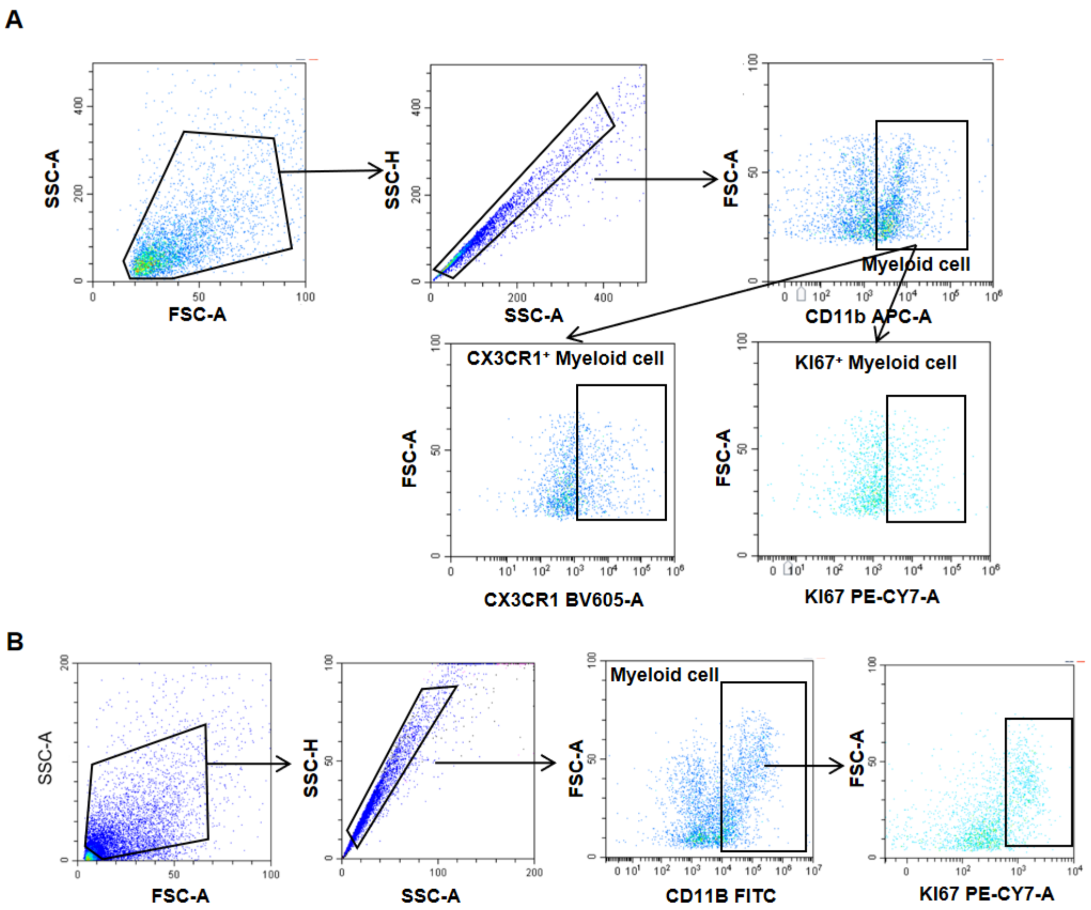

Figure S2. Gating strategy for flow cytometry. (**A**) Gating strategy for flow cytometry analysis of KI67 and CX3CR1 expression in myeloid cells from mouse bone marrow. (**B**) Gating strategy for flow cytometry analysis of KI67 expression in myeloid cell in human skull bone marrow samples.

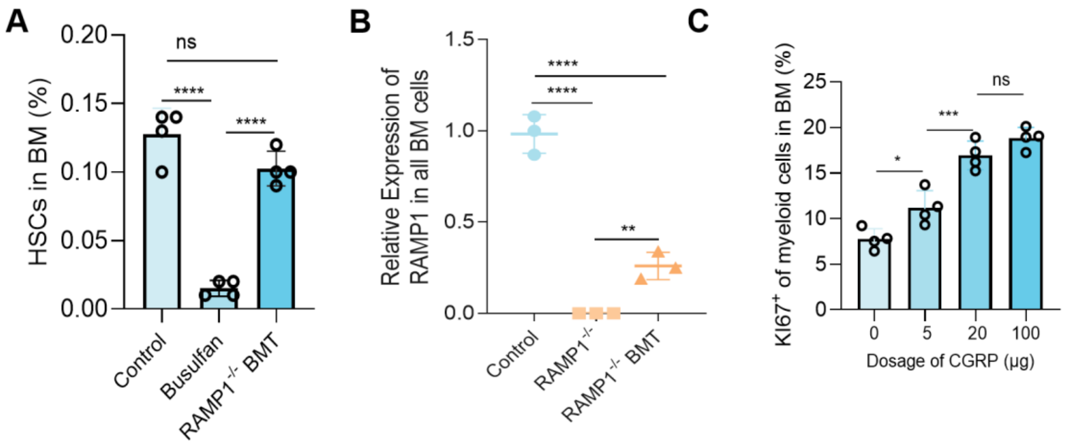

Figure S3. Validation and functional analysis of CGRP-regulated bone marrow myeloid cells. (**A**) Flow cytometry analysis to validate bone marrow cell recovery in chimeric mice after bone marrow transplantation (BMT) (n = 4). (**B**) qPCR analysis of RAMP1 expression in bone marrow cells of chimeric mice, showing significantly reduced RAMP1 expression in the RAMP1-/- BMT group (n = 3). (**C**) CGRP concentration gradient experiment showing that 20 μg CGRP is the optimal concentration, significantly promoting proliferation and migration of CD11b^{+}myeloid cells in the bone marrow (n = 4). Data are shown as mean ± SD. Statistical significance was determined by ANOVA. *P < 0.05, **P < 0.01, ***P < 0.001, ****P < 0.0001.

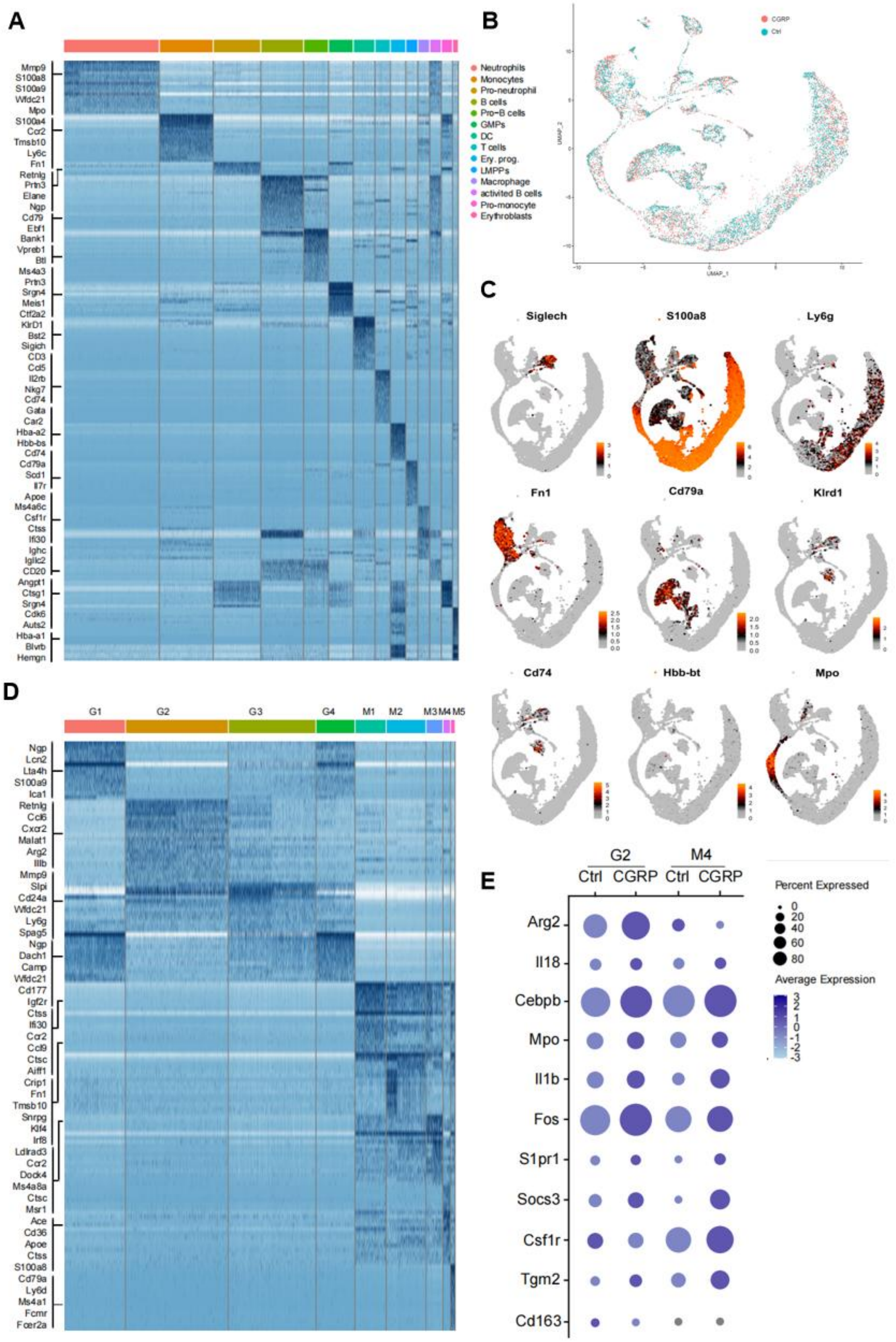

Figure S4. Detailed analysis of single-cell sequencing and functional validation of MDSC. (**A**) Heatmap showing gene expression profiles of clustered cell populations

from single-cell RNA sequencing (scRNA-seq) analysis. (**B**) Overlap plot of cell populations between control and CGRP-treated groups, demonstrating changes in cell composition after CGRP treatment. (**C**) Distribution plot of differentially expressed genes (DEGs), highlighting key marker genes used for single-cell clustering. (**D**) Heatmap showing gene expression profiles of 9 re-clustered myeloid cell subsets. (**E**) Bubble plot showing expression changes of MDSC-related functional genes after CGRP treatment, indicating significantly enhanced immunosuppressive function of MDSC.

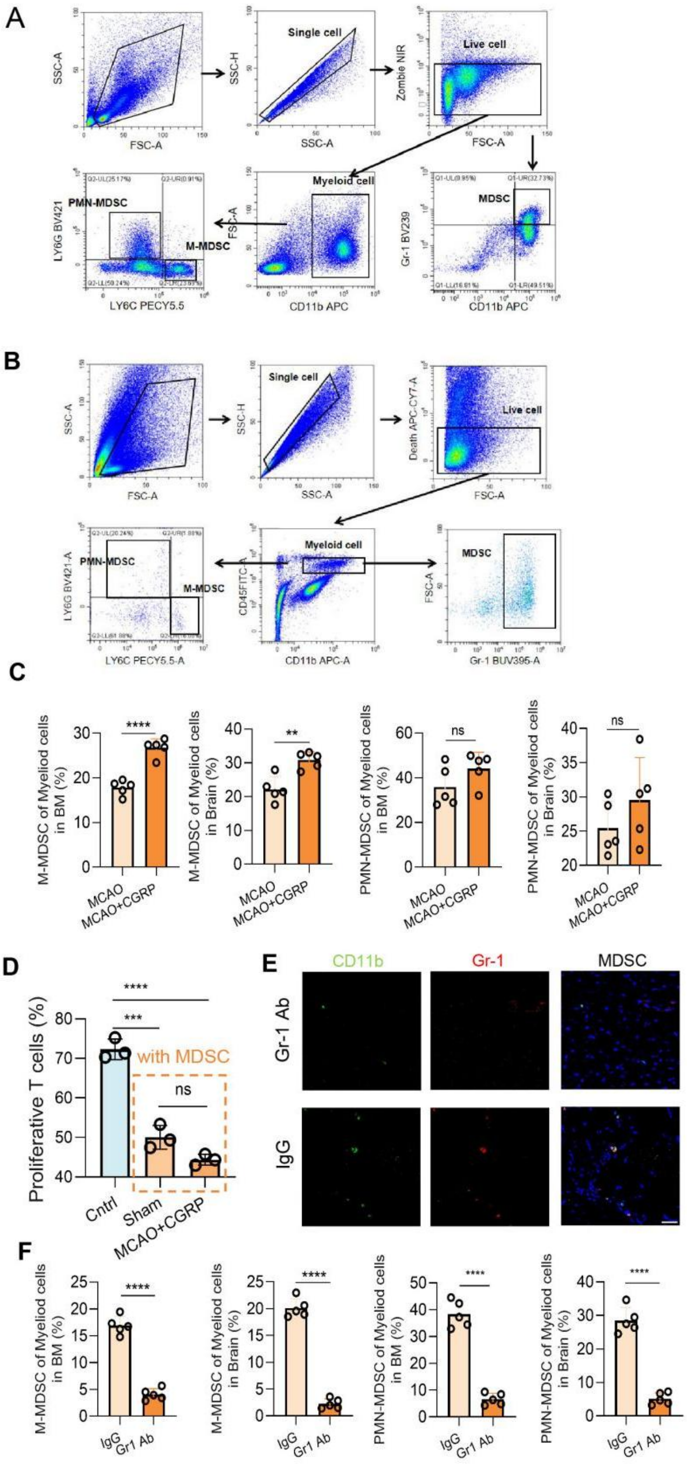

Figure S5. Validation of MDSC depletion and subset analysis. (**A**) Gating strategy for flow cytometry analysis of MDSC subsets in the bone marrow. MDSC are defined

as $CD11b^+Gr\text{-}1^+$ cells, including PMN-MDSC ($CD11b^+Ly6G^+Ly6C^{mid}$) and M-MDSC ($CD11b^+Ly6G^-Ly6C^{hi}$). (**B**) Gating strategy for flow cytometry analysis of MDSC subsets in brain tissues. (**C**) Flow cytometry analysis showing a significant increase in M-MDSC numbers after CGRP treatment, suggesting that CGRP primarily regulates the proliferation and function of M-MDSC (n = 6). (**D**) In vitro suppression assay demonstrating that MDSCs isolated from CGRP-treated brains effectively inhibit T cell proliferation, confirming their immunosuppressive function. (E) Immunofluorescence images validating the effect of Gr-1 antibody-mediated MDSC depletion, showing significantly reduced $CD11b^+Gr\text{-}1^+$ cells in the infarct area after depletion (green: CD11b, red: Gr-1, blue: DAPI for nuclei). The scale bar is 25 μm. (F)Flow cytometric analysis showing that Gr-1 antibody treatment significantly reduced both M-MDSC and PMN-MDSC subsets. Data are shown as mean ± SD. Statistical significance was determined by Student’s t test. $^{**}P < 0.01$, $^{****}P < 0.0001$.

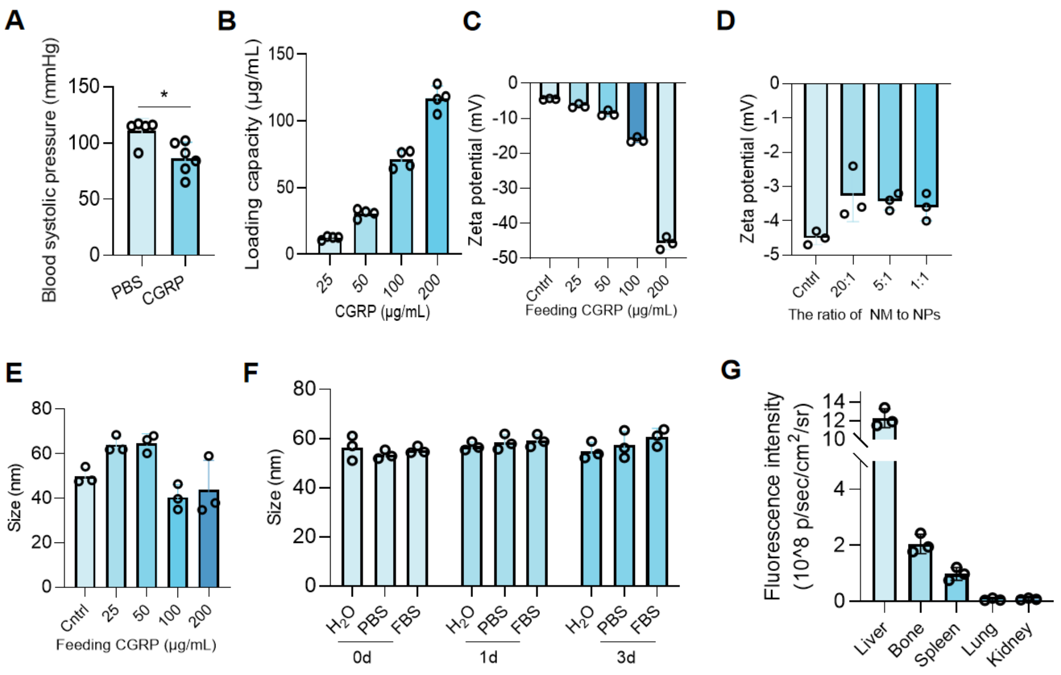

Figure S6. Characterization of nanoparticles. (**A**) Validation of the hypotensive effect of systemic CGRP administration (n = 6). (**B**) UV fluorescence analysis to measure the loading efficiency of CGRP in nanoparticles (n = 4). (**C**) Zeta potential measurement of nanoparticles with different CGRP loading concentrations, showing stable surface charge (n = 3). (**D**) Zeta potential measurement of nanoparticles with different membrane incorporation ratios (n = 3). (**E**) Size distribution analysis of nanoparticles with different CGRP loading concentrations, showing uniform particle size (n = 3). (**F**) Stability test of nanoparticles in different solutions, demonstrating the excellent stability of CGRP-NM@NPs under physiological conditions (n = 5). (G) Biodistribution analysis of CGRP-NM@NPs based on ex vivo fluorescence imaging of the liver, bone, spleen, lungs and kidneys (n = 3). Data are shown as mean ± SD. Statistical significance was determined by Student’s t test. *P < 0.05

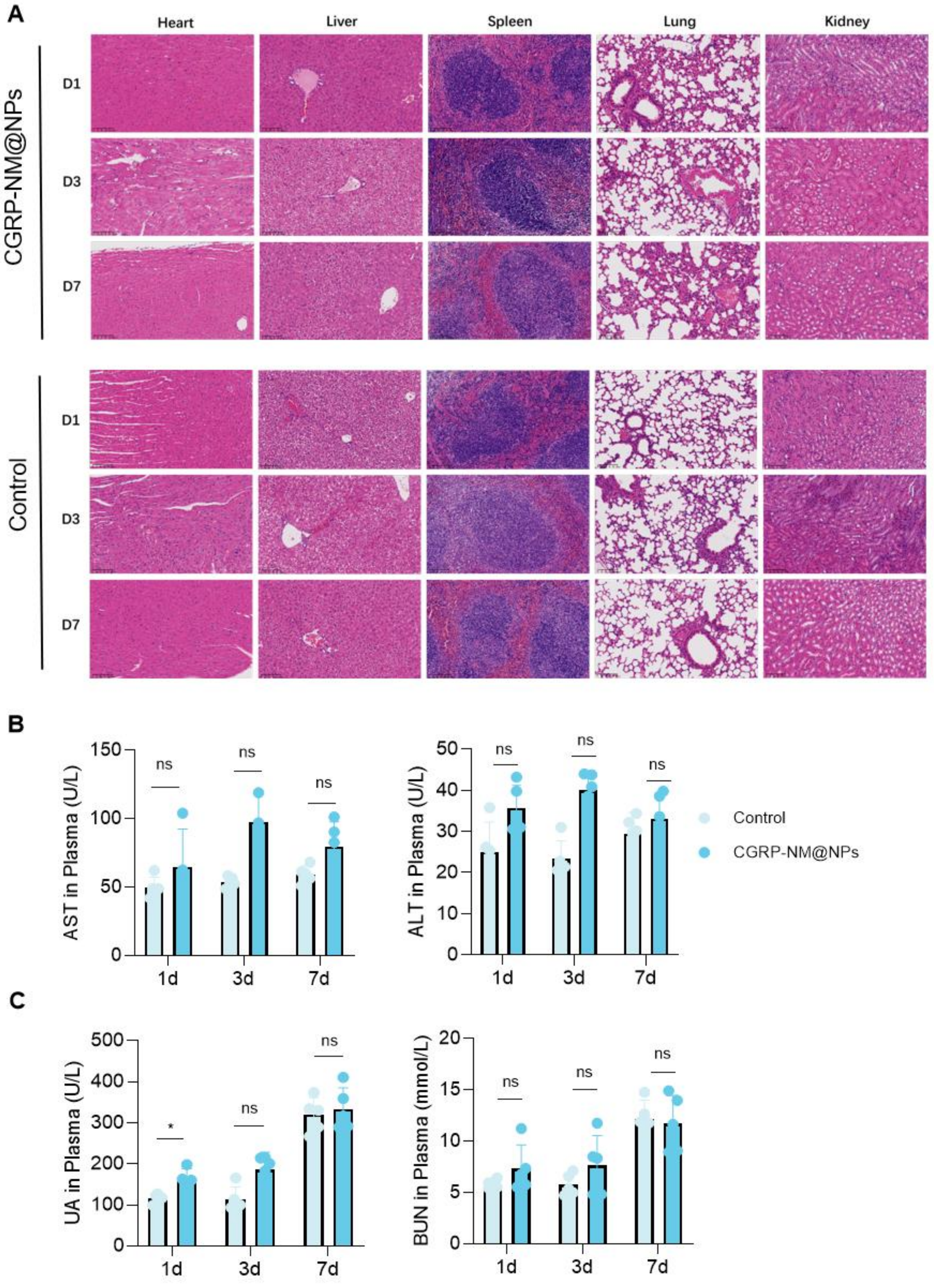

Figure S7. Safety validation of the nanoparticle delivery system. (**A**) Immunohistochemical analysis of major organs (heart, liver, spleen, lung, kidney) showing no significant tissue damage or inflammatory response after CGRP-NM@NPs

treatment. (**B**) Liver function tests (ALT, AST) showing no significant long-term effects of CGRP-NM@NPs treatment on liver function in mice (n = 3). (**C**) Kidney function tests (BUN, UA) showing no significant long-term effects of CGRP-NM@NPs treatment on kidney function in mice (n = 3). Data are shown as mean ± SD. Statistical significance was determined by Student's t test. *$P < 0.05$

Tables S1.Human sample inclusion characteristic.

	Patient ID	Age(years)	Sex	Diagnosis
CONT1	50728887	54	Female	Hemifacial Spasm
CONT2	50760988	55	Female	Hemifacial Spasm
CONT3	50717187	60	Female	Hemifacial Spasm
CONT4	50774326	67	Female	Hemifacial Spasm
CONT5	51083345	59	Male	Hemifacial Spasm
AIS1	15906709	58	Female	Acute Ischemic Stroke
AIS2	51017813	75	Male	Acute Ischemic Stroke
AIS3	60107309	67	Female	Acute Ischemic Stroke
AIS4	15127329	61	Male	Acute Ischemic Stroke
AIS5	9438423	47	Male	Acute Ischemic Stroke
AIS6	51201302	59	Male	Acute Ischemic Stroke

Tables S2.Antibodies used in flow cytometry.

Antibody	Reactivity	Category number	Brand
FITC anti-human CD11b	Human	301329-BLG	BioLegend
PE anti-human CD15 (SSEA-1)	Human	301905-BLG	BioLegend
Brilliant Violet 421™ anti-human HLA-DR	Human	307635-BLG	BioLegend
APC anti-human CD14	Human	367117-BLG	BioLegend
Zombie NIR™ Fixable Viability Kit	Human/Mouse	423106-BLG	BioLegend
PE/Cyanine7 Ki-67	Human/Mouse	652425-BLG	BioLegend
V450 Mouse Lineage Antibody Cocktail	Mouse	51-9006957	BD Biosciences
V450 Mouse Lineage Isotype Control Cocktail	Mouse	51-9006958	BD Biosciences
APC anti-mouse CD117 (c-Kit) Antibody	Mouse	105811-BLG	BioLegend
Brilliant Violet 650™ anti-mouse Sca-1 Antibody	Mouse	108143-BLG	BioLegend
PE anti-mouse CD127 (IL-7R) Antibody	Mouse	135009-BLG	BioLegend
FITC anti - mouse CD45	Mouse	103112-BLG	BioLegend
APC anti - mouse CD11b	Mouse	101212-BLG	BioLegend
Brilliant Violet 421™ anti-mouse Ly-6G	Mouse	127628-BLG	BioLegend
PerCP/Cyanine5.5 anti-mouse Ly-6C	Mouse	128012-BLG	BioLegend
PE anti-mouse Ly-6G/Ly-6C (Gr-1)	Mouse	108407-BLG	BioLegend
Brilliant Violet 605™ anti-mouse IL-10	Mouse	505031-BLG	BioLegend
PE/Cyanine7 anti-mouse CD206 (MMR)	Mouse	141719-BLG	BioLegend
PE anti-mouse CD184 (CXCR4) Antibody	Mouse	146505-BLG	BioLegend
APC/Cyanine7 anti-mouse CD182 (CXCR2) Antibody	Mouse	149313-BLG	BioLegend
Brilliant Violet 605 anti-mouse CX3CR1 Antibody	Mouse	149027-BLG	BioLegend
Fixation Buffer	Mouse	420401-BLG	BioLegend
Intracellular Staining Perm Wash Buffer(10X)	Mouse	421002 -BLG	BioLegend

Tables S3.Primers used for RT-qPCR.

Species	Genes	Forward primer sequence (5'-3')	Reverse primer sequence (5'-3')
Mouse	iNOS	CAAGCACCTTGGAAGAGGAG	AAGGCCAAACACAGCATACC
	CD16	CAGAATGCACACTCTGGAAGC	GGGTCCCTTCGCACATCAG
	CD206	CAAGGAAGGTTGGCATTTGT	CCTTTCAGTCCTTTGCAAGC
	Arg-1	CGCCTTTCTCAAAAGGACAG	CCAGCTCTTCATTGGCTTTC
	β-Actin	AGGCATTGTGATGGACTCCG	AGCTCAGTAACAGTCCGCCTA
	Mpo	AGGGCCGCTGATTATCTACAT	CTCACGTCCTGATAGGCACA
	Il10	TTCTTTCAAACAAAGGACCAGC	GCAACCCAAGTAACCCTTAAAG
	Il6	CTTCTTGGGACTGATGCTGGTGAC	AGGTCTGTTGGGAGTGGTATCCTC
	Il1b	GCAGCAGCACATCAACAAGAGC	AGGTCCACGGGAAAGACACAGG
	S100a8	AAATCACCATGCCCTCTACAAG	CCCACTTTTATCACCATCGCAA
	Stat3	AGGAATCGGCTATATTGCTGGT	CACCTTGGATTGAGAGTCAAGAC
	Hmgb1	CCTTTGATTTTGGGGCGGTA	GCTGACAAGGCTCGTTATGAA
	CCL2	CACTCACCTGCTGCTACTCATTCAC	CTTCTTTGGGACACCTGCTGCTG
	Tgfb1	CCACCTGCAAGACCATCGAC	CTGGCGAGCCTTAGTTTGGAC
	ramp	TCAAAGCACAGTGGTAGCTAGT	ACAGGTGGTCACATGGCATC
Human	β-Actin	CACCAACTGGGACGACAT	ACAGCCTGGATAGCAACG
	IL10	GACTTTAAGGGTTACCTGGGTTG	TCACATGCGCCTTGATGTCTG
	ARG1	GTGGAAACTTGCATGGACAAC	AATCCTGGCACATCGGGAATC
	NOS1	TTCCCTCTCGCCAAAGAGTTT	AAGTGCTAGTGGTGTCGATCT
	S100A8	ATGCCGTCTACAGGGATGAC	ACTGAGGACACTCGGTCTCTA
	STAT3	AAACACCAAAGTGGCATGTGA	AAACACCAAAGTGGCATGTGA
	HIF1A	GAACGTCGAAAAGAAAAGTCTCG	CCTTATCAAGATGCGAACTCACA
	HMGB1	TATGGCAAAAGCGGACAAGG	CTTCGCAACATCACCAATGGA
	TGFB1	GGCCAGATCCTGTCCAAGC	GTGGGTTTCCACCATTAGCAC

Materials and Methods

Flow Cytometry Analysis of Mouse Bone Marrow and Brain Tissue

After euthanasia and perfusion, the right femur was isolated, and bone marrow was collected using a two-chamber centrifugation method: the tibia was cut, placed into a 0.6 mL EP tube, and centrifuged at 2500g for 1 min. Bone marrow was resuspended in 1 mL red blood cell lysis buffer (Thermo Fisher, 00-4333-57, USA) for 5 minutes on ice, filtered through a 70 μm mesh, and terminated with 10 mL PBS. After centrifugation, the pellet was resuspended in 1 mL PBS, yielding ~10^7 cells/tibia. 100 μL was used for flow cytometry.

The left cerebral hemisphere was homogenized on ice, filtered through a 70 μm mesh, and centrifuged at 340 g. The pellet was resuspended in 5 mL 30% Percoll (Cytiva, Sweden) and centrifuged at 900 g for 20 min. The myelin layer was discarded, and the pellet was treated with 200 μL RBC lysis buffer (Thermo Fisher, 00-4333-57, USA) for 5 min on ice, followed by 1 mL PBS and centrifugation at 340 g. The pellet was resuspended in 100 μL PBS for single-cell suspension.

Cells were blocked with 1 μg CD16/CD32 for 20 min at room temperature, stained with viability dye for 10 min on ice, and centrifuged at 600 g. After resuspension in 100 μL flow buffer, cells were stained with flow antibodies for 20 min (antibody details in supplement table), washed, and resuspended in 200 μL flow buffer. For nuclear staining (KI67), cells were permeabilized using FOXP3 Fix/Perm Buffer Set (biolegend,

421403, USA). For intracellular staining (CD206, IL-10), cells were fixed with Intracellular Staining Fixation Buffer (biolegend, 420801, USA) and permeabilized with Permeabilization Wash Buffer (biolegend, 421002, USA). Samples were analyzed using a CytoFLEX flow cytometer (Beckman Coulter, Brea, CA, USA).

Fluorescence Staining and Image Analysis of Mouse Brain Tissue

Brain tissues from mice 48 hours post-cerebral infarction were fixed in 4% PFA, dehydrated in 30% sucrose, and sectioned. Anti-CD11b (MA1-80091,ThermoFisher) and TMEM119 Mouse mAb (98778T,Cell Signaling) at 4 °C overnight. After PBS washing, sections were incubated with Donkey anti-rabbit IgG AF488 (A32790, Invitrogen) and Donkey anti-rat IgG AF594 (A21209, Invitrogen) for 1 h at room temperature in the dark. TUNEL staining was performed using a one-step kit (C1089, Beyotime) according to the manufacturer instructions. Neurons were incubated with Anti-NeuN (104224,Abcam) at 4 °C overnight and incubated with Donkey anti-mouse IgG AF488 (A-21202, Invitrogen)for 1 hour at room temperaturein the dark. Nuclei were counterstained with DAPI, and sections were imaged using a confocal microscope (Leica DMI8). Image J was used for quantitative analysis of immune cell infiltration density and fluorescence intensity in the infarct area. All imaging and analysis were performed in a blinded manner to ensure objectivity.

ELISA Measurement of CGRP

CGRP in bone marrow supernatant was measured using ELISA. Bone marrow cells were centrifuged, and the supernatant was collected and stored at -80°C. Before the experiment, the supernatant was thawed and mixed thoroughly. The RDR-CGRP-Mu Mouse Calcitonin Gene Related Peptide (CGRP) ELISA Kit 96T (ERD202409N5487, RedDot) was used according to the manufacturers' instructions. Absorbance at 450 nm was measured using a microplate reader, and CGRP concentration was calculated based on the standard curve. Each sample was tested in triplicate, and the average value was used as the final result.

RNA Extraction and RT-qPCR

Total RNA was extracted using an RNA extraction kit (Beyotime, R0027, China), and concentration/purity (A260/A280) was measured by NanoDrop. RNA was reverse-transcribed into cDNA using a premix kit (Accurate Biology, AG11728, China). RT-PCR was performed using SYBR Green Premix Pro Taq HS qPCR Kit (Accurate Biology, AG11706, China) on a LightCycler 480 system (Roche, Switzerland). Primer sequences are provided in the Table S3.

MDSC-mediated suppression of T cell proliferation in vitro

MDSC were isolated by fluorescence-activated cell sorting (FACS) based on CD11b and Gr-1 expression. For the sham group, MDSC were collected from the bone marrow of naive C57BL mice. For the experimental group, MDSCs were isolated from the ipsilateral peri-infarct cortex of mice subjected to cerebral ischemia and subsequently

treated with CGRP. Live $CD11b^+Gr\text{-}1^+$ cells were gated and sorted using a FACSAria III cell sorter (BD Biosciences, USA). Splenic T cells were obtained from naive C57BL mice. Single-cell suspensions were prepared by mechanical dissociation of the spleen followed by red blood cell lysis. $CD3^+$ T cells were isolated by FACS using anti-CD3 antibody staining. Purified T cells were labeled with 5 μM CFSE (Thermo Fisher Scientific, USA) .

CFSE-labeled T cells (2×10^5 cells/well) were co-cultured with sorted MDSCs (1×10^5 cells/well) in 96-well flat-bottom plates at a ratio of 2:1. Plates were pre-coated with anti-CD3 antibody (5 μg/ml) in PBS at 37 °C for 2 hours and washed once with PBS before cell seeding. Soluble anti-CD28 antibody (2 μg/ml) was added to each well to provide co-stimulatory signals. Cells were cultured in RPMI 1640 medium supplemented with 10% fetal bovine serum, 1% penicillin/streptomycin, 2 mM L-glutamine, and 50 μM β-mercaptoethanol, in a humidified incubator at 37 °C with 5% CO_2. After 3 days of co-culture, cells were harvested and analyzed by flow cytometry. T cell proliferation was assessed by CFSE dilution using a BD LSRFortessa (BD Biosciences, USA). Live $CD3^+$ T cells were gated, and CFSE fluorescence intensity was analyzed to determine the extent of cell division.

Arterial Pressure Measurement

Arterial blood pressure was non-invasively monitored using a tail-cuff system (Visitech Systems, Apex, BP-2000 series II). Mice were placed on a temperature-controlled platform (37 °C) to promote tail artery dilation and blood circulation. A pressure cuff

connected to a transducer was fitted around the tail. Systolic blood pressures were recorded during gradual cuff deflation following arterial occlusion. Three consecutive measurements were averaged for each recording session. To minimize stress-induced variability, mice were acclimated to the measurement environment for at least 10 min prior to data collection.

Behavioral Assessments

Neurological function was evaluated using the modified Neurological Severity Score (mNSS), a comprehensive assessment scale (0-18 points) encompassing motor, sensory, reflex, and balance functions, with higher scores indicating more severe neurological impairment. The evaluation included specific tests for spontaneous activity, tactile and pain responses, corneal reflex, pinna reflex, and tail flexion reflex.

For sensory-motor asymmetry assessment, the adhesive removal test was performed by applying two 2×3 mm adhesive tapes to the affected forepaw. The time required for tape removal was recorded, with a maximum observation period of 120 s. Each mouse underwent three trials, and the average removal time was calculated. All behavioral tests were independently conducted by two researchers, and the mean values were used for final analysis.

Laser Speckle Contrast Imaging

Regional cerebral blood flow (CBF) was monitored using a laser Doppler flowmetry system (moorVMS-LDF2, Moor Instruments, UK). Following anesthesia, the cranial hair was removed and the skull exposed for imaging. The laser probe was positioned and stabilized to acquire cerebral perfusion images. Image analysis was performed

using PIMSoft software (Perimed, Sweden), with perfusion units (PU) quantified in both the infarct and contralateral regions. The CBF ratio was calculated by comparing perfusion values between these regions.

CGRP Loading Experiment

To covalently conjugate CGRP to PEG, custom NH_2-PEG (Xi'an Ruixi Biotechnology) was used for CGRP modification via amide bond formation. NH_2-PEG and CGRP were mixed at a 1:1 molar ratio in PBS buffer (pH 7.4) and reacted under magnetic stirring in an ice bath for 4 hours. The mixture was then transferred to a dialysis bag (MWCO: 3.5 kDa) and dialyzed against PBS at 4 °C overnight to remove unbound CGRP. The solution inside the dialysis bag was collected as the CGRP-PEG conjugate. To quantify drug loading efficiency, FITC-labeled CGRP (FITC-CGRP, Xi'an Ruixi Biotechnology) was conjugated and purified following the same procedure. The fluorescence intensity (excitation: 490 nm, emission: 520 nm) was measured using a UV-Vis spectrophotometer (UV 2600, Shimadzu), and CGRP concentration and loading efficiency were calculated based on the FITC-CGRP standard curve.

Particle Characterization

The hydrodynamic diameter and zeta potential of biomimetic membrane liposomes were measured using dynamic light scattering (DLS, Zetasizer 3000, Malvern). The liposome solution was diluted with PBS to 0.1-1 mg mL^{-1} and transferred to a cuvette for measurement. Each sample was measured in triplicate, and the average value was recorded. Zeta potential was determined using a dedicated measurement cell, with

triplicate measurements averaged for each sample. The morphology and size of liposomes were further characterized by transmission electron microscopy (TEM, HT-7700, Hitachi).

In Vivo Imaging

To evaluate the in vivo targeting ability of biomimetic membrane liposomes, the hydrophobic fluorescent dye DiD (Beyotime, Cat. CI039) was incorporated into liposomes at a final concentration of 20 μg mL/1. Fluorescently labeled liposomes were injected into mice via the tail vein (100 μL per mouse), and fluorescence intensity in the hind limbs was monitored at 12 h using an in vivo imaging system (PerkinElmer IVIS Spectrum). Prior to imaging, hair was removed from the hind limbs using depilatory cream to minimize background fluorescence. At the time point of peak fluorescence intensity, mice were euthanized, and major organs—including the femur (representing bone marrow), liver, spleen, heart, and lungs—were harvested for ex vivo imaging to assess biodistribution. The fluorescence intensity in each organ was measured, and the bone marrow targeting efficiency was quantified by calculating the fluorescence intensity ratio of the femur to the liver.

Safety Evaluation

Liposomes (100 μL) were injected into mice via the tail vein. Peripheral blood was collected from the tail vein on days 1, 3, and 7 post-injections, and serum was separated for biochemical analysis of liver function markers (ALT, AST) and kidney function markers (BUN, Cr). On day 7, mice were euthanized, and major organs (heart, liver,

spleen, lungs, kidneys) were harvested and fixed in 4% paraformaldehyde for 24 h. The tissues were then paraffin-embedded, sectioned, and stained with hematoxylin and eosin (H&E) for histopathological examination under a light microscope to assess potential tissue damage or inflammatory responses induced by CGRP-NM@NPs.
